# Aberrant GRP78 Phase Transition Sustains Endothelial IRE1α Signaling and Drives Blood–Brain Barrier Failure in Cerebral Amyloid Angiopathy

**DOI:** 10.1002/advs.76761

**Published:** 2026-07-24

**Authors:** Honglin Zheng, Qiang Li, Haiyang Luo, Yapei Yuan, Na Zhang, Yongting Lu, Suying Duan, Jieshi Zhong, Hang Zhang, Chenyang Liu, Yaochong Zhang, Wenzhuo Zhao, Yaxuan Song, Tuo Wang, Si Yan, Yapeng Li, Han Liu, Yuan Gao, Zongping Xia, Yuming Xu

**Affiliations:** ^1^ Department of Neurology the First Affiliated Hospital of Zhengzhou University Zhengzhou University Zhengzhou Henan China; ^2^ NHC Key Laboratory of Prevention and Treatment of Cerebrovascular Disease Zhengzhou Henan China; ^3^ Tianjian Laboratory of Advanced Biomedical Sciences School of Life Sciences Zhengzhou University Zhengzhou Henan China; ^4^ Henan Key Laboratory of Cerebrovascular Diseases Zhengzhou University Zhengzhou Henan China; ^5^ Department of Nephrology The First Affiliated Hospital of Zhengzhou University Zhengzhou University Zhengzhou Henan China; ^6^ Department of Pathology the Second Affiliated Hospital of Zhengzhou University Zhengzhou University Zhengzhou Henan China; ^7^ Clinical Systems Biology Laboratories Translational Medicine Center The First Affiliated Hospital of Zhengzhou University Zhengzhou Henan China

**Keywords:** Aβ40 PFFs, blood–brain barrier, cerebral amyloid angiopathy, endoplasmic reticulum stress, liquid–solid phase transition

## Abstract

Cerebral amyloid angiopathy (CAA) is a major cause of vascular cognitive impairment and lobar intracerebral hemorrhage, yet the mechanisms by which vascular amyloid‐beta (Aβ) disrupts cerebrovascular integrity remain poorly defined. Here, we combined spatiotemporal single‐cell and single‐nucleus transcriptomics in APP23 mice with mechanistic studies in human brain endothelial cells and validation in human CAA‐related hemorrhagic tissues to define the endothelial stress pathways driving disease progression. Endothelial cells emerged as an early and highly vulnerable vascular population during CAA development, characterized by persistent activation of endoplasmic reticulum (ER) stress programs with preferential engagement of the IRE1α branch. In vitro, Aβ40 preformed fibrils directly induced ER remodeling, aberrant solid‐like phase transition of GRP78, sustained IRE1α–TRAF2–JNK signaling, endothelial apoptosis, and tight junction loss. In vivo, pharmacological inhibition of IRE1α with 4µ8C restored junctional integrity, reduced blood‐brain barrier leakage, attenuated vascular amyloid pathology, and improved behavioral performance. Human CAA‐related intracerebral hemorrhage specimens recapitulated endothelial ER stress, apoptotic activation, and tight junction depletion. These findings identify persistent endothelial IRE1α signaling as a key driver of cerebrovascular failure in CAA, highlighting its potential as a therapeutic target for vascular amyloidosis.

## Graphical abstract

Vascular Aβ40 corrupts GRP78 phase behavior in brain endothelial cells, sustaining IRE1α–TRAF2–JNK signaling and driving apoptosis, tight junction loss, and blood–brain barrier failure in cerebral amyloid angiopathy. Pharmacological IRE1α inhibition restores vascular integrity, reduces leakage, and improves functional outcomes, revealing a targetable endothelial stress pathway in vascular amyloidosis.

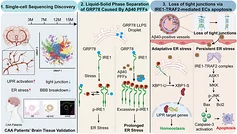

## 1 Introduction

Cerebral amyloid angiopathy (CAA), which is characterized with deposition of amyloid beta 1–40 (Aβ40) in brain vasculature, is the leading cause of vascular dementia and an important comorbidity of Alzheimer's disease (AD) [1, 2, 3]. As one of the most prevalent cerebral small vessel diseases (CSVD), sporadic CAA is found to be present in > 50% of individuals over the age of 80 years [4]. Clinically, advanced CAA is the predominant etiology of spontaneous lobar intracerebral hemorrhage (ICH) and a major catalyst for progressive vascular cognitive impairment [5]. Despite the profound morbidity and mortality, underlying mechanisms of CAA development remain largely elusive, which results in the lack of effective treatment.

The neurovascular unit (NVU), particularly the blood‐brain barrier (BBB), constitutes the primary interface vulnerable to CAA‐induced pathological remodeling [6]. The structural integrity of the BBB is tightly governed by brain endothelial cells and their intercellular tight junction (TJ) networks, encompassing critical transmembrane proteins such as ZO‐1, Occludin, and JAM‐A [7, 8]. Emerging paradigms dictate that BBB dysfunction and ensuing endothelial ablation are not mere terminal sequelae, but rather instigating pathological events that propagate the influx of neurotoxic macromolecules [9], exacerbate localized neuroinflammation, and ultimately precipitate microvascular rupture [10, 11]. Although the accumulation of Aβ40 preformed fibrils (PFFs) is universally acknowledged as a potent vascular stressor, the precise intracellular signal transduction cascades that govern endothelial cell fate and drive BBB hyperpermeability during the spatiotemporal evolution of CAA remain critically unmapped.

Endoplasmic reticulum (ER) stress has emerged as a central pathological determinant in neurodegeneration, orchestrating cellular homeostasis or apoptosis dependent on the chronicity of the insult [12, 13]. Within the unfolded protein response (UPR) network, inositol‐requiring enzyme 1 alpha (IRE1α) functions as a primary and highly conserved stress sensor [14, 15]. While transient IRE1α activation mediates adaptive responses via XBP1 mRNA splicing, sustained and irremediable ER stress triggers paradoxical IRE1α hyperactivation. This chronic state shifts the sensor's conformation, facilitating its direct physical interaction with TNF receptor‐associated factor 2 (TRAF2) [14, 16]. The subsequent assembly of the IRE1α‐TRAF2 signalosome constitutes a lethal apoptotic switch, sequentially recruiting downstream kinases, notably c‐Jun N‐terminal kinase (JNK), to execute caspase‐dependent cellular demise [17, 18]. However, the exact involvement of this specialized, persistent IRE1α‐TRAF2 apoptotic axis in Aβ40‐induced endothelial cytotoxicity and CAA pathogenesis has heretofore eluded characterization.

To address this knowledge gap, we integrated spatiotemporal single‐cell and single‐nucleus transcriptomics, endothelial mechanistic experiments, pharmacological intervention, and human pathological validation to define the stress pathways that drive cerebrovascular failure in CAA. Utilizing high‐resolution single‐cell RNA sequencing (scRNA‐seq) across a comprehensive spatiotemporal continuum (3, 7, 12, and 15 months) in the APP23 transgenic model [9, 19], we systematically mapped the transcriptomic trajectories driving CAA progression, identifying a pronounced and persistent ER stress signature intrinsically enriched within the brain endothelial compartment prior to gross amyloid deposition. Mechanistically, fibrillar Aβ40 promotes aberrant solid‐like phase transition of GRP78, impairs dynamic chaperone behavior, and sustains the IRE1α‐TRAF2‐JNK apoptotic axis, leading to endothelial apoptosis, tight junction loss, and BBB leakage. Pharmacological inhibition of IRE1α with 4µ8C attenuates vascular injury in APP23 mice, while human CAA‐related hemorrhagic tissues recapitulate this endothelial stress‐apoptosis signature. These findings reveal a chaperone‐phase‐dysregulation mechanism linking vascular amyloid stress to BBB failure and support endothelial IRE1α signaling as a candidate therapeutic target in CAA.

## 2 Results

### 2.1 Single‐Cell and Single‐Nucleus Transcriptomics Reveal Cell‐Type‐Specific Transcriptional Remodeling Across CAA Progression

To dissect the cell‐type‐specific transcriptional remodeling associated with CAA progression, we performed single‐cell and single‐nucleus transcriptomic profiling on cortical and vessel‐enriched preparations from APP23 mice and age‐matched wild‐type (WT) littermates at 3, 7, 12 and 15 months of age (Figure 1A). Histopathological analysis revealed a stage‐dependent escalation of amyloid pathology in APP23 mice, with parenchymal Aβ deposition first evident at 7 months, vascular amyloid deposition emerging at 12 months together with increased parenchymal burden, and severe amyloid accumulation in both the cerebrovasculature and parenchyma by 15 months, whereas WT mice showed no detectable amyloid pathology (Figure 1B). In total, we initially captured 359 870 cells and nuclei (Figure S1A–D). Following stringent quality control based on nFeature_RNA, the percentage of mitochondrial transcripts, and the percentage of hemoglobin transcripts, together with doublet removal using DoubletFinder (Figures S1E–L and S2), 288 496 high‐quality cells and nuclei were retained for downstream analyses. Low‐quality profiles were predominantly located at cluster boundaries or formed isolated peripheral groups in the embedding (Figure S1I). We then applied the Harmony algorithm to the filtered dataset to correct batch effects, followed by reclustering, yielding 52 transcriptionally distinct clusters in the integrated atlas (Figure S1M–S). Cells derived from different genotypes, ages and preparations were well intermingled within each annotated population, supporting robust integration across samples (Figure S1Q, R). Based on canonical marker genes, the integrated atlas was annotated into 12 major cell types, including endothelial cells, smooth muscle cells (SMCs), pericytes, microglia, myeloid cells, astrocytes, oligodendrocytes, oligodendrocyte precursor cells (OPCs), glutamatergic neurons, GABAergic neurons, ependymocytes and epithelial cells (Figure 1C, D, and Figure S3). Among the vascular cell populations, we identified endothelial cells, SMCs and pericytes, with endothelial cells representing the predominant vascular cell population in the dataset (45 133 cells), substantially outnumbering SMCs (5722 cells) and pericytes (1826 cells) (Figure 1E, F). The human APP751 transgene was detected across multiple cell types, with the highest expression observed in glutamatergic neurons (Figure S4A, B). Fraction‐resolved composition analysis further showed that E samples were enriched for vascular and glial populations, G samples were predominantly composed of glial cells, and N samples were dominated by neuronal populations (Figure S4C–E). Heatmaps of upregulated and downregulated DEGs across cell types and disease stages showed that transcriptional perturbations were unevenly distributed across the atlas (Figure 1G, H). Astrocytes, microglia and endothelial cells exhibited the largest DEG burden, whereas other cell types showed comparatively fewer changes. The highest numbers of DEGs were observed at 7 and 12 months, with partial overlap in the affected gene sets between these two stages.

**FIGURE 1:**
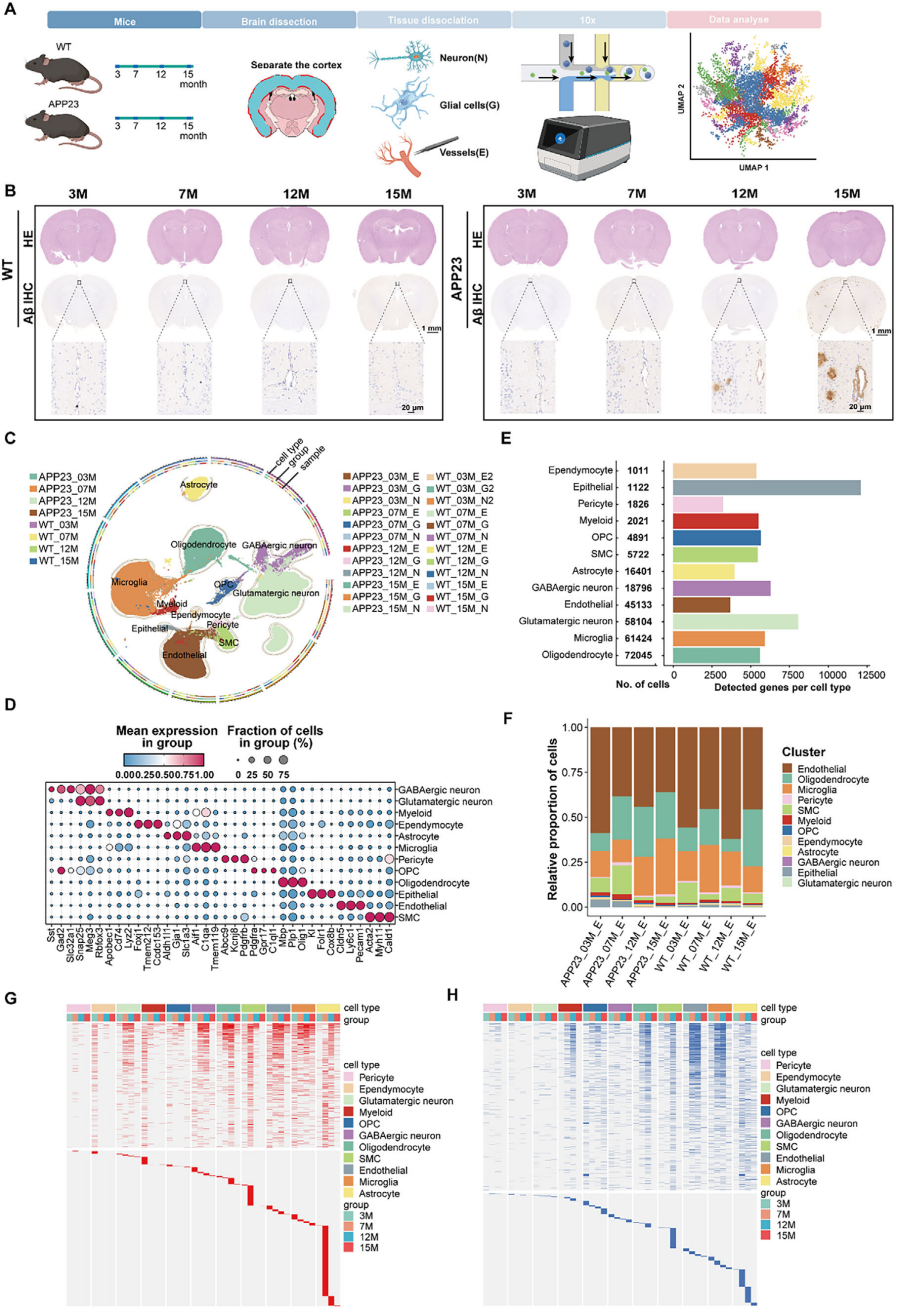
Single‐cell and single‐nucleus transcriptomic profiling reveals cell‐type‐specific remodeling during CAA progression in APP23 mice. (A) Schematic overview of the experimental design and analytical workflow. Cortical tissues were collected from APP23 mice and age‐matched wild‐type (WT) littermates at 3, 7, 12, and 15 months of age, with five mice per genotype at each time point. For each genotype/time‐point group, cerebral cortices from five mice were pooled before tissue dissociation and fractionation. The pooled cortical samples were separated into neuron‐enriched (N), glia‐enriched (G), and vessel‐enriched (E) fractions, followed by single‐cell/single‐nucleus RNA sequencing and downstream integrative analysis. (B) Representative H&E staining and Aβ immunohistochemistry (Aβ IHC) of brain sections from WT and APP23 mice across ages. Scale bars, 1 mm (whole section) and 20 µm (magnified views). (C) Integrated UMAP visualization of all high‐quality cells and nuclei after quality control, reclustering, and batch correction. Outer rings indicate sample identity and fraction type. (D) Dot plot showing canonical marker genes used for cell‐type annotation. Dot size represents the fraction of cells expressing each marker, and color intensity indicates mean expression level. (E) Number of cells and median detected genes per cell for each annotated cell type in the integrated atlas. (F) Stacked bar plots showing the relative cell‐type composition across vessel‐enriched (E) fractions from APP23 and WT mice at 3, 7, 12, and 15 months. (G, H) Heatmaps summarizing significant upregulated (G) and downregulated (H) DEGs across cell types and disease stages in APP23 mice relative to age‐matched WT controls. Significant DEGs were defined as genes with log_2_ fold change > 0.25 for upregulated genes or < −0.25 for downregulated genes, with adjusted *p* value < 0.05. In G, red indicates genes significantly upregulated in APP23 mice relative to age‐matched WT controls; in H, blue indicates genes significantly downregulated in APP23 mice relative to age‐matched WT controls. Gray indicates genes not identified as differentially expressed in the corresponding cell type/time‐point comparison. The upper panels display DEGs shared across multiple cell type/time‐point comparisons, whereas the lower panels display DEGs specific to individual cell type/time‐point comparisons.

We next examined stage‐resolved transcriptional changes in vascular cells, focusing on endothelial cells and SMCs (Figure S5). At 3 months, endothelial cells exhibited a prominent enrichment in pathways related to migration, actin cytoskeleton organization, and tight junctions, which are key to maintaining vascular integrity. Downregulated genes were predominantly associated with protein translation, ribosome biogenesis, and endothelial cell development (Figure S5B). At 7 and 12 months, endothelial cells were characterized by strong enrichment in ribosome‐related pathways and protein synthesis, reflecting the increased metabolic demands associated with vascular remodeling. Concurrently, there was a reduction in genes associated with tight junction assembly and maintenance (Figure S5C, D). By 15 months, endothelial cells showed sustained enrichment for ribosomal and translational programs, indicating ongoing protein synthesis required for stress responses and endothelial dysfunction, while genes involved in actin cytoskeleton and tight junction organization were significantly downregulated (Figure S5E). Consistently, representative stage‐specific DEGs in endothelial cells included Lcn2 at 7 months, Mt1/Mt2 and B2m at 12 months, and Hspb1, Tyrobp and Arhgap15 at 15 months (Figure S5A). At 3 months, SMCs showed significant upregulation of RNA splicing and mRNA metabolic processes, which are crucial for cellular adaptation in response to vascular injury. Downregulated genes were enriched in tight junction and actin filament‐based processes, indicating reduced contractile activity and barrier function (Figure S5F). At 7 and 12 months, SMCs displayed a strong enrichment in ribosome‐, translation‐, and cellular respiration‐related pathways, reflecting an increased need for protein synthesis and energy production. This was coupled with a reduction in tight junction assembly, cell adhesion, and actin cytoskeleton organization, which are critical for maintaining vessel integrity (Figure S5G, H). By 15 months, the altered SMC transcriptome was enriched for regulation of actin filament‐based processes, ECM remodeling, and small GTPase signaling, which are essential for regulating vascular tone and structure. However, the downregulation of genes associated with myofibrils, sarcomere organization, and contractile fibers suggests diminished contractile function and vessel remodeling capacity (Figure S5I).

### 2.2 Endothelial Cells Undergo Stage‐Dependent ER Stress Remodeling Across CAA Progression

Among vascular cell populations, endothelial cells exhibited the largest number of DEGs across CAA progression (Figure 2A). Consistent with this pattern, deconvolution of bulk RNA‐seq data showed that endothelial cells displayed an earlier decline in relative abundance, with a reduction evident from 7 months onward, whereas changes in SMCs and pericytes were less apparent at this stage (Figure 2B and Figure S4F, G).

**FIGURE 2:**
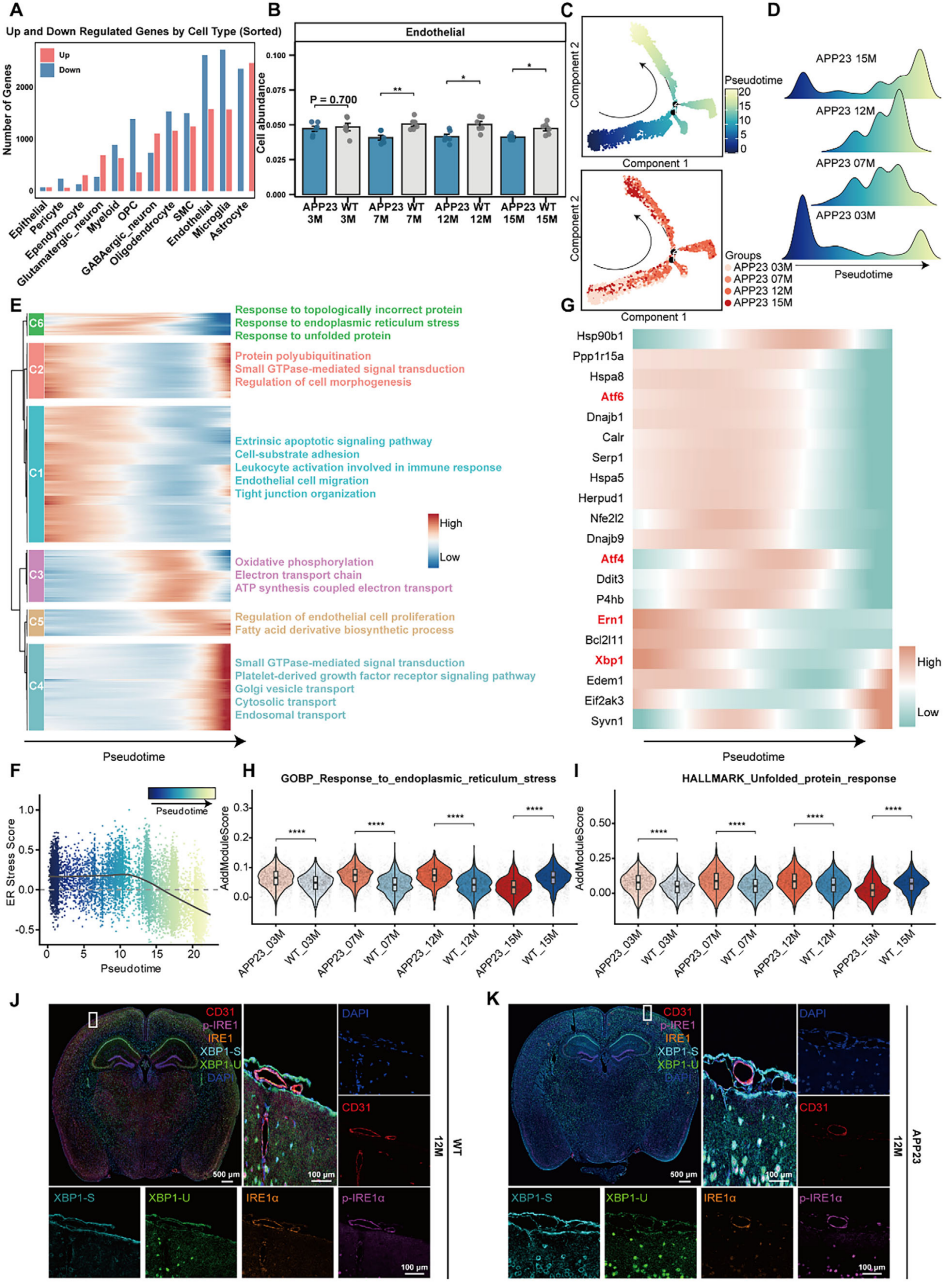
Endothelial cells undergo stage‐dependent ER stress remodeling across CAA progression. (A) Number of upregulated and downregulated DEGs across major cell types, ranked by total DEG burden. (B) Deconvolution analysis of bulk RNA‐seq data showing the relative abundance of endothelial cells in APP23 mice and age‐matched wild‐type (WT) controls at 3, 7, 12, and 15 months. Data are presented as mean ± SEM. Between‐group differences were assessed using Welch's *t*‐test. ^*^ *p* < 0.05, ^**^ *p* < 0.01. (C) Pseudotime trajectory of endothelial cells in APP23 mice. Upper panel, cells were colored by pseudotime. Lower panel, cells were colored by APP23 age group (3, 7, 12, and 15 months). (D) Density distribution of APP23 endothelial cells along pseudotime at different ages. (E) Heatmap of pseudotime‐associated genes grouped into six dynamic expression clusters (C1–C6). Gene expression values were z‐score normalized across pseudotime and displayed as a heatmap, with red indicating high expression and blue indicating low expression. Representative biological processes enriched in each cluster are annotated on the right, selected based on statistical significance (adjusted *P* < 0.01). (F) Endoplasmic reticulum (ER) stress module score plotted along pseudotime. The black line indicates the fitted trend. (G) Heatmap showing the dynamic expression of representative ER stress‐ and unfolded protein response‐related genes along pseudotime. (H, I) Violin plots showing gene set scores for Response to endoplasmic reticulum stress (H) and Unfolded protein response (I) in endothelial cells from APP23 and WT mice at 3, 7, 12, and 15 months. ^****^ *p* < 0.0001 by Wilcoxon rank‐sum test. (J, K) Multiplex immunohistochemistry of brain sections from 12‐month‐old WT (J) and APP23 (K) mice stained for CD31, IRE1α, p‐IRE1α, XBP1‐U, XBP1‐S, and DAPI. Scale bars, 500 µm (whole‐section view) and 100 µm (magnified views).

We next examined the transcriptional dynamics of endothelial cells along disease progression. Pseudotime reconstruction was performed using scRNA‐seq‐derived APP23 endothelial cells from the E/G fractions across 3, 7, 12, and 15 months, with ordering genes selected from age‐matched APP23‐vs‐WT endothelial DEGs to focus on disease‐associated remodeling. This analysis resolved a trajectory originating from a state enriched for 3‐month cells and extending toward later states increasingly populated by endothelial cells from older APP23 mice (Figure 2C, D). To characterize the transcriptional programs underlying this trajectory, pseudotime‐associated genes were grouped into six clusters and visualized by heatmap (Figure 2E). These clusters displayed distinct dynamic patterns along pseudotime and were linked to diverse biological processes, including apoptotic signaling, cell adhesion, immune activation, endothelial migration and tight junction organization (C1); protein polyubiquitination, small GTPase signaling and cell morphogenesis (C2); oxidative phosphorylation and electron transport (C3); vesicle trafficking and intracellular transport‐related pathways (C4); endothelial proliferation and fatty acid derivative biosynthesis (C5); and endoplasmic reticulum stress and unfolded protein responses (C6). Among these clusters, C6 was characterized by progressive upregulation along pseudotime followed by marked suppression at late stages, and was enriched for response to topologically incorrect protein, response to endoplasmic reticulum stress and response to unfolded protein. We next performed gene set scoring to validate the dynamic pattern of C6 along pseudotime. ER stress scores were elevated during the early and intermediate phases of pseudotime, but declined at later pseudotime states (Figure 2F). Consistent with this pattern, the expression of representative ER stress‐related genes also changed dynamically along the trajectory (Figure 2G). *Ern1* and *Xbp1* reached peak expression at early pseudotime and then gradually declined, whereas *Atf6* showed a broadly similar trend with a smaller dynamic range. In contrast, *Atf4* showed an increase at intermediate pseudotime states.

To distinguish the effects of disease progression from those of aging alone, we further compared endothelial cells between APP23 and WT mice at matched ages. Gene set scoring showed that ER stress‐ and UPR‐related signatures were significantly higher in APP23 endothelial cells than in WT controls at 3, 7 and 12 months, but significantly lower at 15 months (Figure 2H, I and Figure S6 A, B). Branch‐specific scoring further resolved distinct temporal patterns across the three canonical UPR arms (Figure S7). IRE1‐related signatures were elevated earlier during disease progression, with higher scores in APP23 endothelial cells than in age‐matched WT controls from 3 months onward (Figure S7 A, B), whereas PERK‐related signatures became elevated later and were mainly increased at 12 and 15 months (Figure S7 C, D). By contrast, ATF6‐related changes were comparatively limited, with only modest alterations detected at late stages (Figure S7 E, F). Consistent with the early engagement of the IRE1 branch, *Hspa5* expression remained significantly elevated in APP23 endothelial cells across all examined time points, whereas *Xbp1* expression was significantly altered relative to WT controls (Figure S7G, H). Multiplex immunofluorescence (mIHC) further validated activation of the IRE1‐XBP1 axis in cerebrovascular endothelial cells (Figure 2J, K and Figure S8). In 12‐month‐old APP23 mice, CD31‐positive endothelial cells lining cerebral vessels exhibited stronger p‐IRE1α and XBP1‐S signals than those in age‐matched WT mice (Figure 2J, K). Analysis across different ages revealed that IRE1α‐XBP1 signaling remained largely unchanged in APP23 mice at 3 and 7 months of age compared with age‐matched WT controls, but was significantly elevated in 12‐month‐old APP23 mice (Figure S8).

### 2.3 Aβ40 PFFs Directly Instigate Structural ER Remodeling and Persistent IRE1α Hyperactivation in human Brain Endothelial Cells

To establish whether Aβ40 accumulation is the direct instigator of the endothelial ER stress observed in our single‐cell transcriptomic analysis and validated by in vivo mIHC, we transitioned to an in vitro model using the human brain microvascular endothelial cell line, hCMEC/D3. Cells were treated with varying concentrations (0 to 20 µm) of Aβ40 PFFs. Immunoblotting revealed a striking, dose‐dependent upregulation of the master UPR chaperone GRP78. Concomitantly, the IRE1α axis was robustly activated, evidenced by a dramatic increase in p‐IRE1α and its downstream active transcription factor, XBP1‐S (Figure 3A, Figure S9A–E). These biochemical alterations plateaued at higher concentrations, indicating a profound and sustained UPR engagement.

**FIGURE 3:**
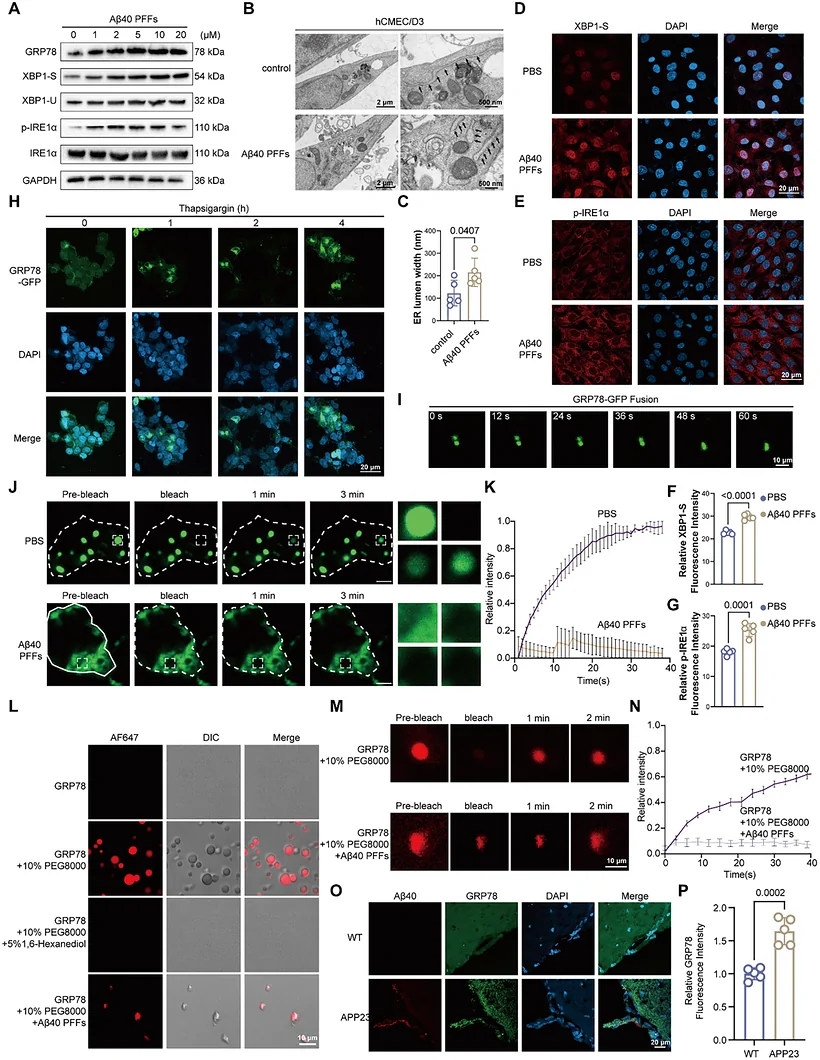
Aβ40 PFFs induce severe endothelial ER stress and drive an aberrant solid‐like phase transition of GRP78. (A) Representative immunoblots of GRP78, XBP1‐S, XBP1‐U, p‐IRE1α, IRE1α, and GAPDH in hCMEC/D3 cells treated with increasing concentrations of Aβ40 preformed fibrils (PFFs) (0, 1, 2, 5, 10, and 20 µm). (B, C) Representative transmission electron microscopy (TEM) images (B) and quantification of ER lumen width (C) in hCMEC/D3 cells exposed to vehicle control or Aβ40 PFFs. Black arrows indicate ER structures. Scale bars, 2 µm and 500 nm. (D–G) Representative immunofluorescence images and corresponding quantifications of XBP1‐S (D, F) and p‐IRE1α (E, G) in hCMEC/D3 cells treated with or without Aβ40 PFFs. Nuclei were counterstained with DAPI. Scale bars, 20 µm. (H) Time‐course imaging of GRP78‐GFP in hCMEC/D3 cells following thapsigargin treatment, showing the formation of GRP78‐positive condensates. Nuclei were stained with DAPI. Scale bar, 20 µm. (I) Time‐lapse imaging capturing the fusion events of GRP78‐GFP droplets in living cells, a biophysical hallmark of liquid–liquid phase separation (LLPS). Scale bar,10 µm. (J, K) Cellular fluorescence recovery after photobleaching (FRAP) analysis. (L) In vitro cell‐free droplet formation assay using purified recombinant His‐GRP78. (M, N) Fluorescence recovery after photobleaching (FRAP) imaging of recombinant GRP78 condensates. (O) Immunofluorescence staining of Aβ40, GRP78, and DAPI in brain sections from WT and APP23 mice. Scale bar, 20 µm. (P) Quantification of GRP78 fluorescence intensity from (O). Data are presented as mean ± SEM.

To ascertain whether these biochemical signatures reflect actual organelle damage, we evaluated the ultrastructure of the ER using transmission electron microscopy (TEM). While vehicle‐treated control cells exhibited normal, tightly packed ER networks, exposure to Aβ40 PFFs triggered severe morphological remodeling. The ER compartments in Aβ40 PFFs‐treated cells displayed massive luminal dilation and vacuolization (Figure 3B, C), an unequivocal ultrastructural hallmark of extreme, unresolvable ER stress. Corroborating the immunoblotting data, immunofluorescence analysis further confirmed the robust intracellular aggregation of p‐IRE1α and the prominent nuclear translocation of XBP1‐S in the presence of Aβ40 PFFs (Figure 3D–G).

To determine whether the endothelial ER stress response was specifically dependent on the fibrillar conformation of Aβ40 rather than nonspecific peptide exposure, we next generated a fibrillization‐defective Aβ40 double mutant, Aβ40 (L17A/F19A) [20, 21]. Transmission electron microscopy and atomic force microscopy confirmed that, unlike WT Aβ40, the L17A/F19A mutant failed to form typical fibrillar assemblies (Figure S10A, B). Functionally, treatment with the mutant species induced markedly weaker ER stress responses in hCMEC/D3 cells, as evidenced by reduced upregulation of GRP78, p‐IRE1α and XBP1‐S compared with WT Aβ40 PFFs (Figure S11 A–F, J–M). Consistent with this blunted stress activation, the mutant also exhibited a substantially diminished capacity to suppress the tight junction proteins Occludin, JAM‐A and ZO‐1 (Figure S11 G–I). These findings indicate that the ability of Aβ40 to trigger persistent endothelial ER stress and junctional disruption is critically dependent on its fibrillar assembly state.

### 2.4 Aβ40 PFFs Drive an Aberrant Solid‐Like Phase Transition of GRP78 to Sustain IRE1α Hyperactivation

Under physiological steady‐state conditions, the ER chaperone GRP78 binds to the luminal domain of IRE1α, acting as an endogenous repressor to maintain it in an inactive conformation [22, 23]. Upon encountering proteotoxic stress, GRP78 dissociates from IRE1α, triggering the UPR [24, 25]. Recent studies suggest that GRP78 undergoes liquid‐liquid phase separation (LLPS) to form dynamic condensates, which serve as transient microcompartments to sequester and process misfolded amyloidogenic proteins [25, 26, 27].

Building upon the recent paradigm that endoplasmic reticulum stress induces liquid–liquid phase separation of GRP78 and modulates protein aggregation dynamics, we hypothesized that Aβ40 PFFs might hyperactivate IRE1α by perturbing this critical phase separation process. To empirically test the biophysical state and dynamics of GRP78, we employed Fluorescence Recovery After Photobleaching (FRAP) utilizing a GRP78‐GFP fusion construct. While classical ER stress induced by thapsigargin resulted in the expected formation of GRP78 droplets with rapid fluorescence recovery, reflecting a healthy, liquid‐like molecular mobility (Figure 3H, I), challenge with Aβ40 PFFs severely abrogated this dynamic behavior. Following photobleaching, the fluorescence recovery of GRP78‐GFP in Aβ40 PFF‐treated cells was drastically blunted and incomplete compared to controls (Figure 3J, K). This profound loss of intra‐droplet fluidity strongly implies an aberrant phase transition from highly dynamic liquid condensates to solid‐like pathological aggregates.

To establish whether the aberrant phase transition of GRP78 is a direct biophysical consequence of Aβ40 encounter rather than an indirect cellular stress response, we reconstituted the phase behavior in a highly controlled, cell‐free system (Figure 3L, Figure S9F). Under physiological macromolecular crowding conditions, purified recombinant His‐GRP78 spontaneously underwent liquid‐liquid phase separation (LLPS), forming highly dynamic, spherical droplets. Strikingly, co‐incubation with Aβ40 PFFs directly corrupted the physical state of these pure GRP78 condensates, driving a morphological shift from spherical droplets into irregular, amorphous aggregates.

To quantitatively profile this rheological shift, we performed in vitro fluorescence recovery after photobleaching (FRAP) assays (Figure 3M, N). While the pure GRP78 condensates exhibited rapid and robust fluorescence recovery characteristic of liquid‐like internal molecular diffusion, the presence of Aβ40 PFFs severely abrogated this intra‐droplet mobility. The fluorescence recovery was nearly entirely stalled, confirming a transition into a solid‐like state. Consequently, Aβ40 PFFs effectively trap GRP78 in a solid‐like state, persistently preventing it from re‐associating with and inhibiting IRE1α, thereby locking the downstream apoptotic signaling in a state of chronic hyperactivation.

To further examine whether extracellular fibrillar Aβ40 could access intracellular compartments relevant to GRP78‐associated ER stress, we performed confocal imaging using AF488‐labeled Aβ40 PFFs in hCMEC/D3 cells. Aβ40 PFFs‐AF488 were detected as intracellular puncta, and orthogonal Z‐stack reconstruction confirmed that these signals were localized within the cellular volume rather than merely attached to the cell surface (Figure S12A, B). ER‐Tracker co‐staining and line‐scan analysis showed spatial proximity between internalized Aβ40 PFFs and ER‐associated signals (Figure S12C, D). Moreover, GRP78 immunofluorescence revealed close apposition or partial spatial association between Aβ40 PFFs‐AF488 puncta and GRP78‐positive structures, whereas the fibrillization‐defective Aβ40(L17A/F19A) mutant showed weaker intracellular signal and reduced association with ER/GRP78‐associated regions (Figure S12C–E). These findings provide endothelial‐cell‐based evidence supporting the cellular relevance of the GRP78 phase‐transition mechanism observed in the cell‐free assay.

Finally, to bridge these rigorous in vitro mechanisms back to the in vivo CAA pathology, we assessed GRP78 expression within the cerebral vasculature of APP23 mice. Strikingly consistent with our cellular models, robust GRP78 immunoreactivity was exclusively enriched and heavily colocalized with vascular Aβ deposits in the brain sections of APP23 mice, whereas it was barely detectable in age‐matched WT controls (Figure 3O, P). Collectively, these multi‐modal findings unequivocally demonstrate that perivascular Aβ40 directly provokes profound ER structural deterioration and locks the endothelial IRE1α signaling pathway in a persistent state of hyperactivation.

### 2.5 Endothelial ER Stress Is Coupled to Tight Junction Disassembly and Blood‐brain Barrier Leakage During CAA Progression

To determine whether the sustained endothelial ER stress identified above was accompanied by structural deterioration of the cerebrovascular barrier, we next interrogated endothelial junction‐related programs across CAA progression. Stratification of endothelial cells according to ER stress activity revealed a transcriptional shift tightly coupled to barrier disruption. Endothelial cells with high ER stress scores exhibited marked upregulation of stress‐associated genes, including canonical unfolded protein response and oxidative stress‐related transcripts, whereas genes involved in tight junction assembly, actin filament organization, cell adhesion and cell–cell junction maintenance were prominently suppressed (Figure 4A, B). Module scoring further showed that the overall tight junction transcriptomic signature in APP23 endothelial cells differed from that in WT controls in a stage‐dependent manner, being higher at 3 and 7 months but lower at 12 and 15 months (Figure 4C, Figure S6C). Cell–cell interaction analysis revealed that endothelial‐associated communication in APP23 mice became progressively dysregulated compared with WT mice, with an overall reduction in interaction strength across disease progression (Figure 4D, Figures S13 and S14). Notably, several endothelial communication pairs associated with barrier integrity and vascular homeostasis became selectively undetectable at later disease stages. The tight junction‐related pair Ocln–Ocln was lost in APP23 mice at 12 and 15 months, whereas Jam2–F11r and F11r–Jam2 were lost at 15 months. In addition, Jag2–Notch1 was lost at 12 and 15 months, and Lamb2–(Itga1+Itgb1) was lost at 15 months in APP23 mice (Figure 4E and Figure S15).

**FIGURE 4:**
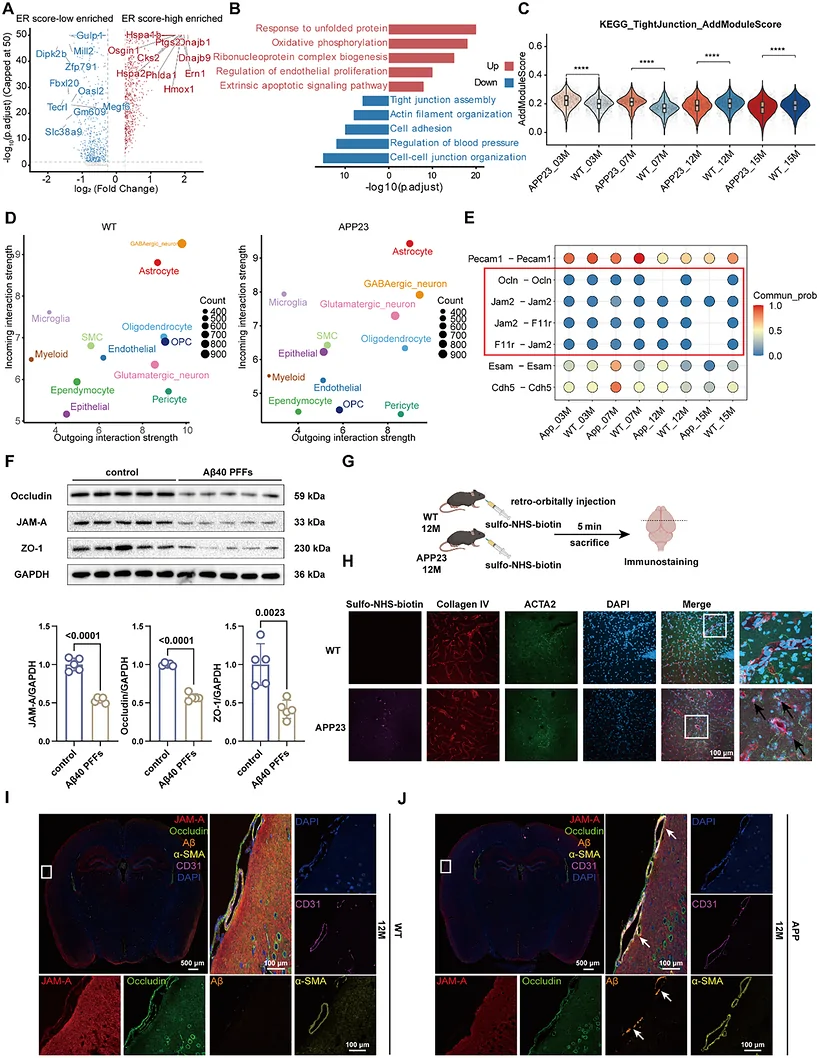
Persistent endothelial ER stress is associated with tight junction disassembly and blood–brain barrier leakage during CAA progression. (A) Volcano plot showing differentially expressed genes between ER score‐low‐enriched and ER score‐high‐enriched endothelial cells. Top 10 genes enriched in the ER score‐high group and ER score‐low group are indicated. (B) Bar plot showing GO enrichment analysis results for differentially expressed genes in ER stress score‐high vs. score‐low endothelial cells. Enriched terms are shown separately for upregulated (red) and downregulated (blue) genes. (C) Violin plots showing tight junction gene set scores in endothelial cells from APP23 and age‐matched wild‐type (WT) mice at 3, 7, 12, and 15 months. ^****^ *p* < 0.0001 by Wilcoxon rank‐sum test. (D) Cell–cell communication analysis showing incoming and outgoing interaction strength of major cell types in WT and APP23 mice. Dot size indicates the number of interactions. (E) The plot shows the communication probabilities (Commun_prob) of representative ligand‐receptor pairs in APP23 and WT mice across different ages. Dot color intensity corresponds to the communication level. The red box highlights representative endothelial communication pairs related to barrier integrity and vascular homeostasis. The absence of a dot indicates no detectable communication. (F) Representative immunoblots and quantification of JAM‐A, Occludin, ZO‐1, and GAPDH in hCMEC/D3 cells treated with or without Aβ40 PFFs. Data are presented as mean ± SEM. (G) Schematic illustration of the in vivo sulfo‐NHS‐biotin permeability assay. Twelve‐month‐old WT and APP23 mice received retro‐orbital injection of sulfo‐NHS‐biotin, followed by tissue collection 5 min later for immunostaining analysis. (H) Representative images of sulfo‐NHS‐biotin permeability assay in WT and APP23 mice. Arrows indicate tracer leakage into the surrounding parenchyma. Scale bar, 100 µm. (I, J) Multiplex immunohistochemistry of brain sections from 12‐month‐old WT (I) and APP23 (J) mice stained for JAM‐A, Occludin, Aβ, α‐SMA, CD31, and DAPI. Arrows indicate Aβ‐positive perivascular deposits in APP23 brain sections. Scale bars, 500 µm (whole‐section view) and 100 µm (magnified views).

To validate these transcriptomic observations in a reductionist system, we challenged hCMEC/D3 cells with Aβ40 PFFs and assessed core tight junction proteins. Immunoblotting demonstrated that Aβ40 PFF exposure significantly decreased the expression of Occludin, JAM‐A and ZO‐1, confirming that fibrillar Aβ40 directly compromises endothelial junctional integrity in vitro (Figure 4F) [28].

We next sought to confirm this structural and functional vascular collapse in vivo. To directly assess BBB permeability, we performed an in vivo sulfo‐NHS‐biotin tracer assay via retro‐orbital injection in 12‐month‐old mice [29], a stage characterized by established vascular amyloidosis (Figure 4G). While the biotin tracer remained strictly confined within the vessel lumen delineated by Collagen IV and ACTA2 in WT mice, APP23 mice exhibited massive microvascular leakage, with the tracer profoundly extravasating into the surrounding brain parenchyma (Figure 4H). Finally, comprehensive mIHC on 12‐month‐old brain sections mapped this barrier failure directly to Aβ pathology. In WT brain sections, Aβ staining was largely absent, and the cerebrovasculature exhibited continuous and intact tight junction networks, as indicated by Occludin and JAM‐A staining (Figure 4I). In contrast, APP23 mice showed prominent perivascular Aβ deposition, as indicated by arrows, together with marked fragmentation and reduction of Occludin and JAM‐A in Aβ‐burdened vascular regions (Figure 4J). Analysis of earlier time points revealed a similar stage‐dependent pattern. While junctional architecture was largely preserved at 3 months, APP23 mice began to exhibit visible discontinuity of Occludin and JAM‐A labeling along CD31‐positive vessels by 7 months compared with WT controls (Figure S16), indicating that endothelial junction disorganization emerges progressively during CAA development. Collectively, these data establish that Aβ‐induced ER stress is inextricably linked to the physical dismantling of tight junctions and the ultimate functional failure of the BBB in CAA.

### 2.6 Persistent ER Stress Drives Endothelial Apoptosis Through the IRE1α‐TRAF2‐JNK‐Caspase‐3 Axis

To further clarify how sustained endoplasmic reticulum stress leads to tight junction disruption during CAA progression, we next investigated whether endothelial cell death constitutes the direct structural basis for barrier failure. Multiplex immunohistochemistry of cell death‐related markers showed that cleaved Caspase‐3 was markedly increased in 12‐month‐old APP23 mice compared with age‐matched WT controls. p‐RIPK1 signals were also increased in APP23 mice, whereas GSDMD‐NT and p‐MLKL showed no significant differences between groups [30, 31](Figure 5A, Figure S17). Given that cleaved Caspase‐3 displayed a more prominent increase and represents a canonical executioner of apoptosis, we focused our subsequent analyses on Caspase‐3‐associated apoptotic signaling in Aβ40‐induced cerebrovascular injury. These findings indicate that endothelial loss in CAA is predominantly associated with apoptotic execution rather than alternative lytic death programs. Consistent with this observation, flow cytometric analysis in hCMEC/D3 cells demonstrated that Aβ40 PFFs exposure significantly increased the proportion of apoptotic cells compared with untreated controls (Figure 5B). To determine whether this apoptotic tendency was also reflected at the transcriptomic level in vivo, we interrogated apoptosis‐related gene programs in endothelial cells from APP23 mice across disease progression. Gene set scoring showed that intrinsic apoptotic signaling was consistently elevated in APP23 endothelial cells relative to age‐matched WT controls, supporting the existence of a sustained pro‐apoptotic state during CAA development (Figure 5C).

**FIGURE 5:**
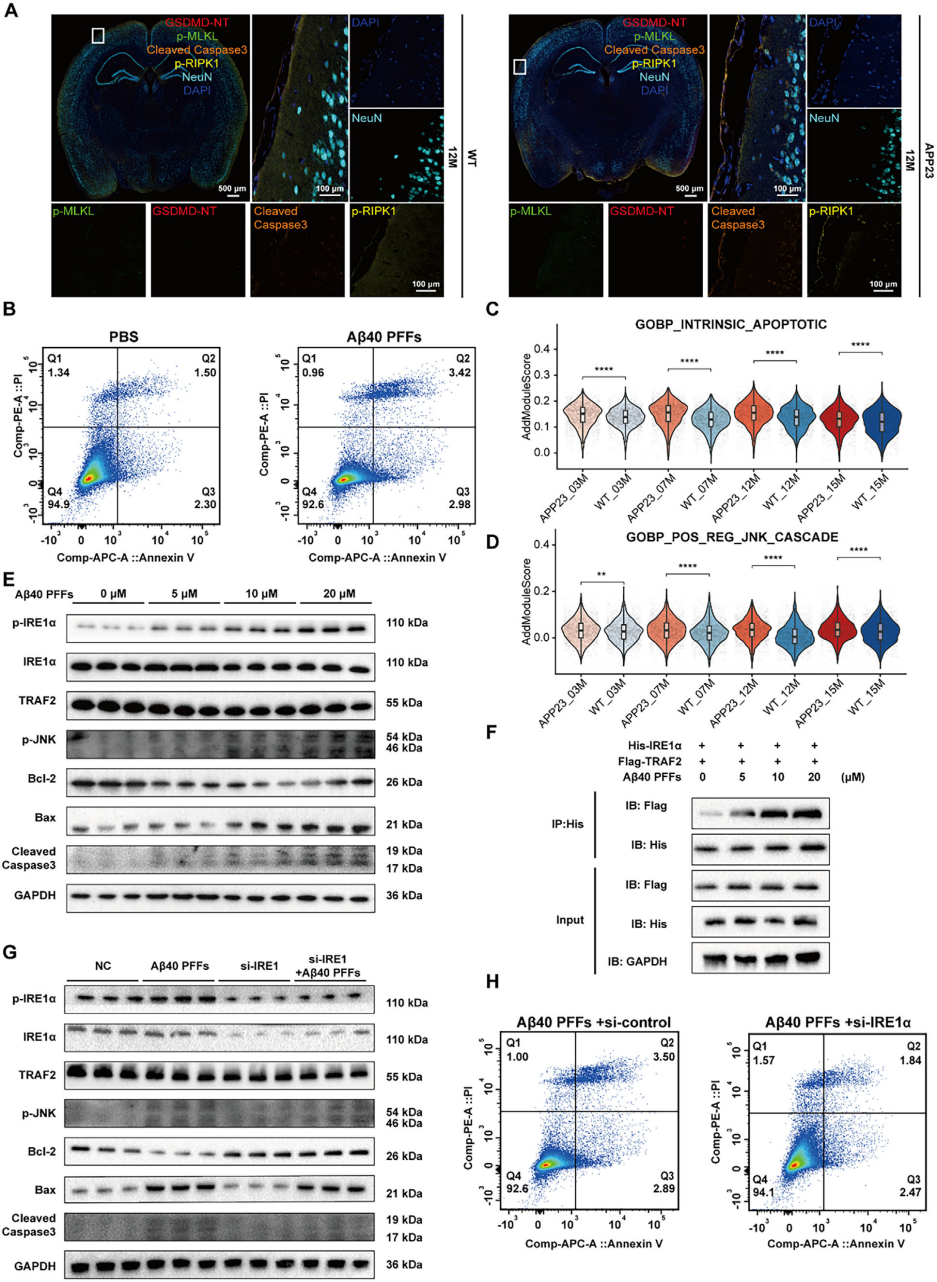
Persistent ER stress drives endothelial apoptosis through the IRE1α–TRAF2–JNK–caspase‐3 axis. (A) Multiplex immunohistochemistry of brain sections from 12‐month‐old wild‐type (WT) and APP23 mice stained for p‐RIPK1, p‐MLKL, GSDMD‐NT, cleaved Caspase‐3, NeuN, and DAPI. Scale bars, 500 µm (whole‐section view) and 100 µm (magnified views). (B) Representative flow cytometry plots of Annexin V/PI staining in hCMEC/D3 cells treated with or without Aβ40 PFFs. (C, D) Violin plots showing gene set scores for intrinsic apoptotic (C) and positive regulation of JNK cascade (D) in endothelial cells from APP23 and age‐matched wild‐type (WT) mice at 3, 7, 12, and 15 months. ^**^ *p* < 0.01, ^****^ *p* < 0.0001 by Wilcoxon rank‐sum test. (E) Representative immunoblots of p‐IRE1α, IRE1α, TRAF2, p‐JNK, Bcl‐2, Bax, cleaved Caspase‐3, and GAPDH in hCMEC/D3 cells treated with increasing concentrations of Aβ40 PFFs (0, 5, 10, and 20 µm). (F) Co‐immunoprecipitation analysis of His‐tagged IRE1α and Flag‐tagged TRAF2 in the presence of increasing concentrations of Aβ40 PFFs. (G) Representative immunoblots of p‐IRE1α, IRE1α, TRAF2, p‐JNK, Bcl‐2, Bax, cleaved Caspase‐3, and GAPDH in hCMEC/D3 cells transfected with control siRNA or siRNA targeting IRE1α and treated with or without Aβ40 PFFs. (H) Representative flow cytometry plots of Annexin V/PI staining in hCMEC/D3 cells treated with Aβ40 PFFs following transfection with control siRNA or siRNA targeting IRE1α.

To mechanistically delineate how Ern1 connects to this apoptotic outcome in endothelial cells, we performed an in silico virtual knockout of Ern1 using scTenifoldKnk, applied to single‐cell RNA sequencing data from vascular endothelial cells of 12‐month‐old APP23 mice. This approach constructs a single‐cell gene regulatory network and quantifies the transcriptional perturbation of each gene upon removal of Ern1, yielding 46 significantly perturbed genes (*p* < 0.05, FDR < 0.10; Figure S18A). Pathway and process enrichment analysis was performed on these perturbed genes across multiple ontology sources, and enriched terms were clustered by membership similarity and rendered as a network plot (Figure S18D). The resulting network revealed convergence on several functionally coherent clusters, prominently featuring inflammatory and immune signaling, regulation of apoptotic pathways, JNK cascade activation, and vascular development, collectively suggesting that Ern1 occupies a central regulatory position linking ER stress to endothelial cell death and vascular remodeling programs in CAA.

To further characterize the broader transcriptional perturbation landscape, we performed GSEA on the full ranked gene list from the virtual knockout output, ordered by perturbation Z‐score. Representative enrichment results across eight biological themes—including translation and ribosome biogenesis, mitochondrial oxidative phosphorylation, MAPK/JNK signaling, apoptosis, UPR/ER stress, neuroinflammation, blood–brain barrier and endothelial function, and vascular remodeling—are summarized in Figure S18B, highlighting the broad regulatory impact of Ern1 on CAA‐relevant pathways. Among these, JNK signaling‐related gene sets were consistently and significantly enriched. Focused GSEA for the GO Biological Process term “Positive Regulation of JNK Cascade” confirmed robust activation (NES = 2.269, FDR = 8.83 × 10^−^^7^; Figure S18C), suggesting that Ern1 may actively promote JNK pathway upregulation in the CAA endothelium. Corroborating this computational prediction, gene set scoring across four disease progression time points in APP23 endothelial cells demonstrated that positive regulation of the JNK cascade was consistently elevated relative to age‐matched WT controls (Figure 5D), providing in vivo transcriptomic evidence that Ern1‐driven JNK activation is a sustained feature of CAA pathogenesis.

Given the established role of IRE1α in coupling chronic ER stress to death signaling [32], we next examined the IRE1α‐TRAF2‐JNK apoptotic axis in vitro. Immunoblotting revealed that Aβ40 PFFs triggered a dose‐dependent increase in p‐IRE1α and p‐JNK, whereas total TRAF2 protein levels remained largely unchanged. Concurrently, Aβ40 PFFs induced a marked reduction in the anti‐apoptotic protein Bcl2, although this decrease did not follow a strict dose‐dependent pattern. Bax and cleaved Caspase‐3 were increased, particularly at higher Aβ40 PFF concentrations (Figure 5E, Figure S19). Co‐immunoprecipitation further demonstrated that Aβ40 PFFs enhanced the physical association between IRE1α and TRAF2, indicating assembly of the pro‐death IRE1α‐TRAF2 signaling complex under amyloid stress (Figure 5F).

To directly test whether this pathway is required for Aβ40‐induced endothelial apoptosis, we silenced IRE1α in hCMEC/D3 cells prior to Aβ40 PFFs challenge. IRE1α knockdown attenuated Aβ40 PFF‐induced activation of the downstream apoptotic pathway, as reflected by reduced p‐JNK, partial restoration of Bcl‐2, significant attenuation of Bax upregulation, and decreased cleaved Caspase‐3 levels (Figure 5G, Figure S20). Functionally, flow cytometry further confirmed that IRE1α silencing significantly decreased the proportion of apoptotic cells following Aβ40 PFFs treatment (Figure 5H). Collectively, these results demonstrate that persistent ER stress drives endothelial apoptosis predominantly through the IRE1α‐TRAF2‐JNK‐caspase‐3 axis, thereby providing a mechanistic explanation for the progressive loss of tight junction integrity and cerebrovascular barrier breakdown in CAA.

### 2.7 Pharmacological Inhibition of IRE1α Signaling Restores Tight Junction Networks and Abrogates Blood–Brain Barrier Breakdown

To determine whether targeted inhibition of the IRE1α axis could serve as a viable therapeutic strategy against Aβ‐induced endothelial damage, we first evaluated the efficacy of 4µ8C, a highly specific IRE1α RNase domain inhibitor [18, 33], in our in vitro hCMEC/D3 cell model. Following Aβ40 PFFs challenge, concurrent treatment with 4µ8C effectively attenuated the pathological ER stress response. Quantitative immunoblotting confirmed that 4µ8C intervention significantly attenuated GRP78 upregulation, suppressed IRE1α phosphorylation, and robustly blocked downstream XBP1 splicing (Figure 6A–F). Crucially, this targeted pharmacological blockade successfully rescued endothelial barrier components, restoring the expression of essential tight junction proteins, including Occludin, JAM‐A, and ZO‐1, to near‐physiological levels (Figure 6A, G–I).

**FIGURE 6:**
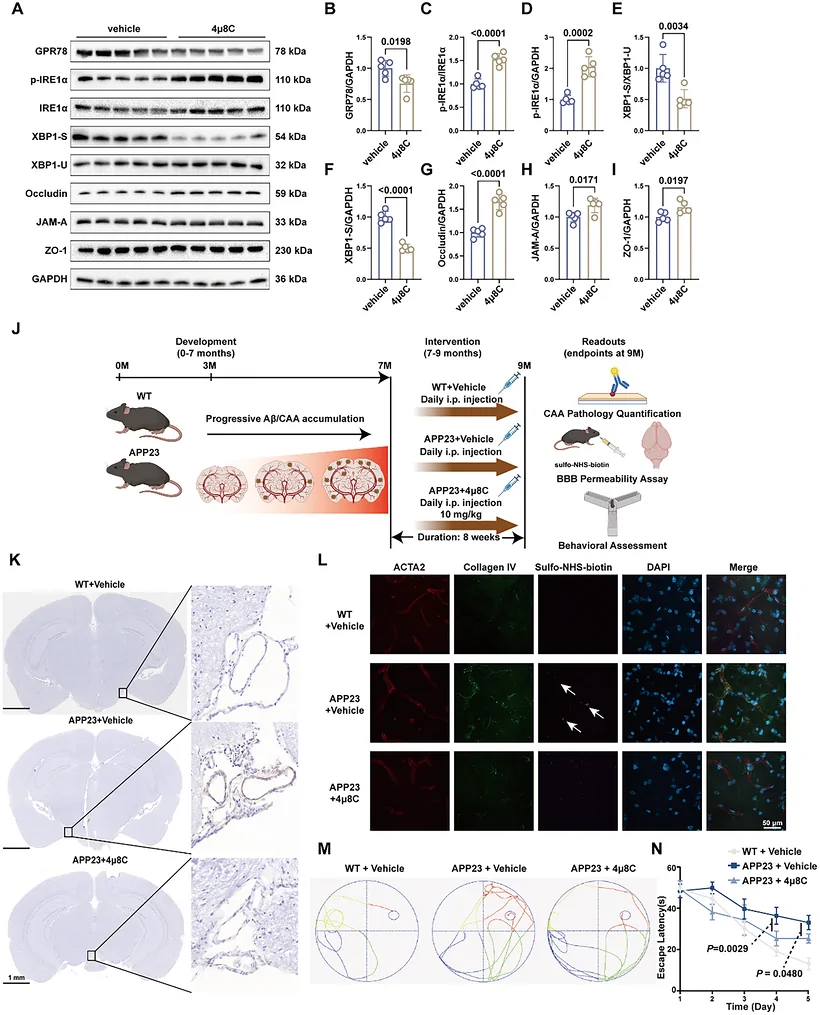
Pharmacological inhibition of IRE1α signaling restores endothelial tight junctions, reduces blood‐brain barrier leakage, and improves functional outcomes during CAA progression. (A) Representative immunoblots of GRP78, p‐IRE1α, IRE1α, XBP1‐S, XBP1‐U, Occludin, JAM‐A, ZO‐1, and GAPDH in hCMEC/D3 cells treated with Aβ40 preformed fibrils (PFFs) in the presence of vehicle or the IRE1α RNase inhibitor 4µ8C. (B–I) Quantification of GRP78/GAPDH (B), p‐IRE1α/IRE1α (C), p‐IRE1α/GAPDH (D), XBP1‐S/XBP1‐U (E), XBP‐S/GAPDH (F), Occludin/GAPDH (G), JAM‐A/GAPDH (H), ZO‐1/GAPDH (I). (J) Schematic illustration of the prophylactic in vivo intervention paradigm. (K) Representative Aβ immunohistochemistry of brain sections from WT + Vehicle, APP23 + Vehicle, and APP23 + 4µ8C mice. Scale bar, 1 mm. L) Representative images of the in vivo sulfo‐NHS‐biotin permeability assay in WT + Vehicle, APP23 + Vehicle, and APP23 + 4µ8C mice. Arrows indicate extravascular sulfo‐NHS‐biotin tracer leakage signals. Scale bar, 50 µm. (M) Representative swimming trajectories from behavioral testing in WT + Vehicle, APP23 + Vehicle, and APP23 + 4µ8C mice. (N) Quantification of escape latency during training days in the indicated groups. Data are presented as mean ± SEM.

Encouraged by these robust in vitro protective effects, we next sought to validate the translational potential of 4µ8C in vivo. Based on our spatiotemporal histopathological and transcriptomic profiling, 7 months of age represented an early transition window during APP23 disease progression, when parenchymal Aβ deposition had emerged and endothelial ER stress/IRE1‐XBP1‐related programs were already altered, whereas overt vascular Aβ deposition and severe tight junction collapse became more prominent at later stages (Figure 1B, G, H; Figure 2H, I; Figure 4C; Figure S6; Figure S7A–F). We therefore designed a prophylactic intervention paradigm starting at 7 months of age. Seven‐month‐old APP23 mice and their WT littermates were administered daily intraperitoneal (i.p.) injections of 4µ8C (10 mg/kg) or vehicle for 8 consecutive weeks (Figure 6J).

We first investigated whether systemic IRE1α inhibition translated into tangible protection against cerebrovascular pathology. Histological evaluation via Aβ IHC demonstrated that chronic 4µ8C treatment notably ameliorated the severity of CAA, with treated mice exhibiting a visible reduction in vascular amyloid burden compared to their vehicle‐treated counterparts (Figure 6K). Because IRE1α signaling is positioned downstream of Aβ40‐induced endothelial stress, we further examined whether this reduction could be attributed to altered APP expression. Quantitative PCR and immunoblotting showed that 4µ8C treatment did not significantly reduce APP mRNA or APP protein levels in APP23 mice compared with vehicle‐treated APP23 mice (Figure S21). These data suggest that the reduced vascular Aβ40 burden observed after 4µ8C treatment is unlikely to be primarily explained by decreased APP expression.

Most importantly, to ascertain the functional integrity of the BBB, we performed an in vivo sulfo‐NHS‐biotin permeability assay. In vehicle‐treated APP23 mice, the cerebrovasculature was profoundly compromised, characterized by massive extravasation of the biotin tracer (magenta) beyond the basement membrane (Collagen IV) and smooth muscle coverage (ACTA2) deep into the brain parenchyma. In stark contrast, in vivo 4µ8C treatment markedly reduced this microvascular leakage, keeping the biotin tracer tightly restricted within the vessel lumen (Figure 6L). To further determine whether preservation of cerebrovascular integrity translated into functional benefit, we next assessed cognitive performance by behavioral testing. Representative swimming trajectories showed that APP23 mice treated with vehicle displayed disorganized search strategies compared with WT controls, whereas 4µ8C‐treated APP23 mice exhibited a visibly improved navigation pattern (Figure 6M). Consistent with this observation, APP23 mice in the vehicle group showed a prolonged escape latency across training days, indicative of impaired spatial learning, while 4µ8C treatment significantly shortened the escape latency and partially rescued the learning deficit (Figure 6N). These findings suggest that pharmacological inhibition of IRE1α not only mitigates vascular and barrier pathology, but also confers measurable behavioral improvement during CAA progression.

Collectively, these in vitro and in vivo findings provide convergent evidence that pharmacological inhibition of the IRE1α stress axis attenuates Aβ‐associated tight junction disruption, reduces BBB leakage, and partially improves functional outcomes during CAA progression.

### 2.8 Human CAA‐Related Intracerebral Hemorrhage Recapitulates Endothelial ER Stress, Tight Junction Depletion, and Apoptotic Activation

To definitively establish the clinical relevance and translational validity of our experimental findings, we sought to verify whether the specific pathological triad observed in our murine and cellular models, including endothelial ER stress, tight junction depletion, and subsequent apoptosis, is recapitulated in human CAA. We procured perihematomal brain tissues and hematoma specimens from patients undergoing emergent surgical evacuation for spontaneous ICH. Crucially, we compared samples from patients diagnosed with CAA‐related ICH against those with hypertensive ICH, utilizing the latter as a disease‐matched, non‐amyloid hemorrhagic control (Figure 7A, Figure S22).

**FIGURE 7:**
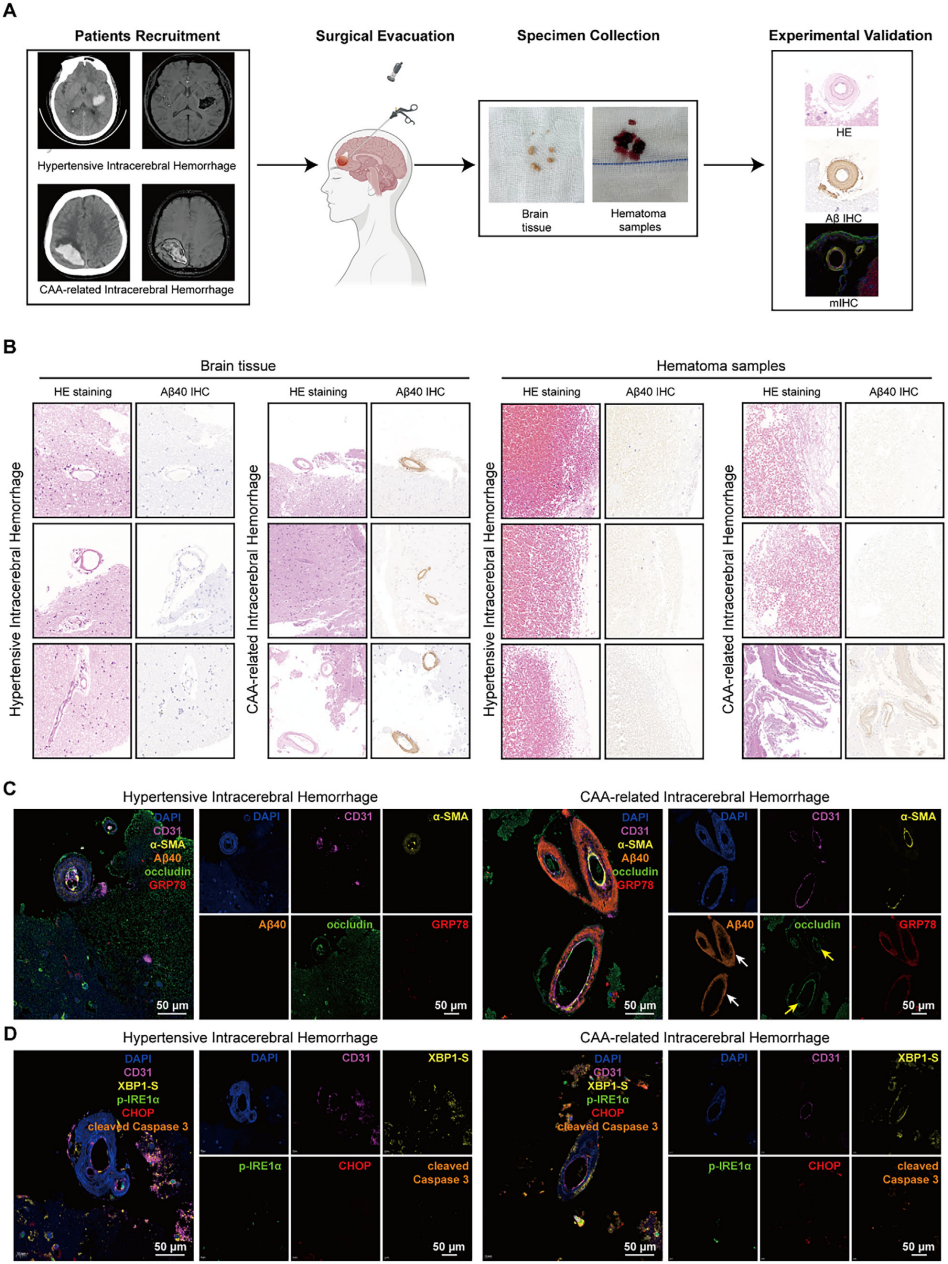
Human CAA‐related intracerebral hemorrhage recapitulates endothelial ER stress, tight junction depletion, and apoptotic signaling observed in experimental models. (A) Schematic overview of the clinical sample collection and validation workflow. Patients with hypertensive intracerebral hemorrhage or CAA‐related intracerebral hemorrhage underwent surgical evacuation, followed by collection of perihematomal brain tissue and hematoma specimens for histological and multiplex immunohistochemical analyses. (B) Representative H&E staining and Aβ40 IHC of perihematomal brain tissue and hematoma samples from patients with hypertensive intracerebral hemorrhage and CAA‐related intracerebral hemorrhage. (C) Multiplex immunohistochemistry of human brain sections from hypertensive intracerebral hemorrhage and CAA‐related intracerebral hemorrhage stained for CD31, α‐SMA, Aβ40, Occludin, GRP78, and DAPI. White arrows indicate Aβ40 deposits, and yellow arrows indicate areas showing loss of Occludin staining. Scale bars, 50 µm. (D) Multiplex immunohistochemistry of human brain sections from hypertensive intracerebral hemorrhage and CAA‐related intracerebral hemorrhage stained for CD31, XBP1‐S, p‐IRE1α, CHOP, cleaved Caspase‐3, and DAPI. Scale bars, 50 µm.

Initial histopathological evaluation using HE and Aβ40 immunohistochemistry confirmed the distinct vascular etiologies of the two cohorts. While both groups exhibited severe tissue disruption characteristic of ICH, Aβ40 deposition was strictly localized within the blood vessel walls of the CAA‐related ICH samples, and was completely absent in the hypertensive ICH specimens (Figure 7B).

We next utilized mIHC on these clinical specimens to precisely map the spatial relationship between vascular amyloid burden, ER stress, and BBB architecture. In the hypertensive ICH cohort, despite the occurrence of vascular rupture, the expression of the UPR master chaperone GRP78 remained low within the CD31‐positive endothelial layer, and the tight junction protein Occludin was relatively preserved. In stark contrast, the cerebrovasculature of CAA patients was heavily encased by Aβ deposits. In CAA‐related ICH samples, vascular Occludin staining showed a reduced and discontinuous pattern compared with hypertensive ICH controls, suggesting impaired tight junction integrity in Aβ‐burdened cerebrovascular regions. (Figure 7C).

Finally, to verify if this ER stress ultimately translates into the specific apoptotic execution we delineated in vitro, we probed the human tissues for the IRE1α terminal signaling cascade. Remarkably mirroring our murine and cellular data, the endothelial compartment in CAA‐related ICH exhibited robust hyperactivation of p‐IRE1 and significant nuclear accumulation of spliced XBP1 (XBP1‐S). This persistent UPR signaling engaged the pro‐apoptotic transcription factor CHOP, ultimately culminating in the pronounced activation of cleaved Caspase‐3 within the CD31‐positive cells (Figure 7D). Conversely, this apoptotic axis was quiescent in the hypertensive ICH vessels.

Collectively, these human pathological signatures support the clinical relevance of our mechanistic model. It is demonstrated that the Aβ‐driven, IRE1α‐mediated endothelial apoptotic switch is not merely an experimental phenomenon, but a clinically relevant mechanism contributing to tight junction degradation and cerebrovascular collapse in CAA.

## 3 Discussion

In the present study, we identify persistent endothelial ER stress as a central pathogenic event in CAA and demonstrate that the IRE1α–TRAF2 signaling axis functions as a critical molecular switch linking vascular Aβ40 deposition to endothelial apoptosis, tight junction breakdown, and blood–brain barrier failure. Crucially, we uncover a novel biophysical paradigm wherein fibrillar Aβ40 drives an aberrant solid‐like phase transition of the chaperone GRP78, physically stabilizing this stress signaling. By integrating spatiotemporal single‐cell transcriptomics across an unprecedented 15‐month continuum in APP23 mice with mechanistic validation in hCMEC/D3 cells and pathological confirmation in human CAA‐related ICH tissues, we establish a coherent disease framework. Our data suggest that endothelial dysfunction in CAA is not merely a secondary consequence of advanced vascular fragility, but rather an actively regulated, structure‐dependent, and therapeutically targetable execution program.

A major finding of this study is that endothelial cells exhibit the most prominent and earliest transcriptional remodeling among vascular cell populations during CAA progression. Compared with smooth muscle cells and pericytes, endothelial cells showed the largest differential gene burden and an earlier decline in relative abundance, indicating that the endothelial compartment may represent one of the first vascular barriers destabilized during disease evolution [34]. This observation is particularly important because CAA has traditionally been interpreted largely through the lens of amyloid deposition and vessel wall fragility [1, 2, 35]. Our results extend this view by showing that, even before overt end‐stage vascular collapse, endothelial cells undergo a progressive shift toward stress‐associated, pro‐apoptotic, and junction‐disruptive states. Thus, the vascular pathology of CAA should be considered not only a problem of extracellular amyloid accumulation, but also a problem of maladaptive endothelial stress signaling and cellular fate conversion.

Among the unfolded protein response branches, our data point to a preferential and relatively early engagement of the IRE1 pathway in endothelial cells. Both single‐cell scoring and in situ validation showed that IRE1‐related signatures were elevated from early stages of disease progression, whereas PERK activation appeared later and ATF6‐related changes were comparatively limited. This branch‐specific pattern suggests that endothelial cells in CAA do not mount a generic or uniform UPR response, but instead undergo a selective stress adaptation program that progressively shifts toward IRE1α‐centered signaling [36, 37]. Such temporal selectivity is mechanistically meaningful. IRE1α is well known to function as a bifunctional sensor capable of supporting adaptive homeostasis under transient stress, but of promoting cell death under chronic and unresolved proteotoxic conditions [12, 38, 39]. Our findings indicate that in the context of vascular Aβ40 accumulation, endothelial IRE1α is not simply activated, but persistently hyperactivated, thereby favoring transition from an initially compensatory response toward a maladaptive pro‐death program [40, 41].

Another important contribution of this study is the demonstration that fibrillar Aβ40 itself is sufficient to directly trigger severe ER remodeling and sustained IRE1α activation in brain endothelial cells. In hCMEC/D3 cells, Aβ40 PFFs induced dose‐dependent upregulation of GRP78, phosphorylation of IRE1α, XBP1 splicing, and striking ER luminal dilation and vacuolization, indicating that the endothelial ER is a direct intracellular target of vascular amyloid stress. Notably, this pathogenic effect depended strongly on the fibrillar assembly state of Aβ40, since the fibrillization‐defective L17A/F19A mutant showed markedly attenuated stress‐inducing and junction‐disruptive activity [20, 21]. This observation strengthens the biological relevance of fibrillar Aβ40 as a disease‐driving conformer in CAA and argues against the possibility that the observed endothelial toxicity simply reflects nonspecific peptide exposure. Rather, our data support a structure‐dependent mechanism whereby fibrillar Aβ40 exerts a qualitatively distinct proteotoxic pressure on endothelial cells.

Our study further provides a biophysical layer of mechanistic explanation by linking Aβ40‐induced ER stress to aberrant phase behavior of GRP78. Under physiological stress adaptation, GRP78 is expected to dynamically dissociate from IRE1α and participate in liquid‐like condensates that help buffer proteotoxic burden. However, under Aβ40 PFF challenge, GRP78 displayed markedly impaired FRAP, consistent with a transition toward a less dynamic, solid‐like state. Based on these findings, we propose that fibrillar Aβ40 not only activates ER stress, but also corrupts a key adaptive buffering mechanism by immobilizing GRP78 and preventing re‐establishment of proper IRE1α restraint. In this model, persistent IRE1α activation is sustained not merely by the presence of misfolded protein, but by a failure of chaperone‐phase dynamics required for stress resolution. This concept may be particularly relevant to chronic protein misfolding disorders [42, 43], in which maladaptive transitions of stress‐response condensates could amplify and stabilize otherwise transient signaling states.

Functionally, our data position endothelial apoptosis as the structural event that translates intracellular stress into barrier failure. Transcriptomic analyses showed that high‐ER‐stress endothelial states were accompanied by suppression of tight junction assembly, cell adhesion, and actin organization programs, whereas in vivo and in vitro experiments confirmed depletion of Occludin, JAM‐A, and ZO‐1 together with overt BBB leakage. We further show that Aβ40 PFFs activate the IRE1α–TRAF2–JNK cascade, reduce Bcl‐2, increase Bax and cleaved Caspase‐3, and enhance apoptosis in endothelial cells, while IRE1α silencing substantially reverses these effects. These results support a model in which tight junction disruption in CAA is not solely due to passive junctional remodeling, but is closely coupled to progressive endothelial cell loss through apoptosis. The relative absence of comparable activation of necroptotic or pyroptotic markers in our experimental setting further suggests that apoptotic execution is a dominant mode of endothelial demise downstream of chronic Aβ40‐induced ER stress.

The translational relevance of this mechanism is reinforced by our human tissue analyses. In perihematomal tissues from patients with CAA‐related ICH, we observed the same pathological triad identified in our experimental systems: vascular Aβ accumulation, strong endothelial ER stress signaling, and severe tight junction depletion accompanied by apoptotic activation. The fact that these features were not comparably present in hypertensive ICH controls argues that the observed endothelial IRE1α‐associated stress signature is not simply a nonspecific consequence of hemorrhage or tissue injury, but is closely related to amyloid vasculopathy itself [44]. This human validation is particularly important because it bridges a common gap in vascular amyloid studies, where mechanistic observations in animal or cell models often remain difficult to anchor in genuine clinical pathology. This is consistent with extensive evidence showing robust UPR activation in the context of human Alzheimer's disease pathology [45]. Our findings therefore support the notion that the Aβ‐driven endothelial stress‐apoptosis program described here is not merely an experimental artifact, but a clinically relevant component of human CAA.

From a therapeutic perspective, our data identify IRE1α as a potentially actionable target in CAA. Pharmacological inhibition with 4µ8C effectively suppressed endothelial stress signaling, restored tight junction proteins, reduced vascular amyloid‐associated barrier leakage, and partially improved behavioral performance in APP23 mice. These findings suggest that modulation of IRE1α may confer benefit not only by attenuating intracellular stress signaling, but also by preserving cerebrovascular integrity and, secondarily, mitigating functional decline. Notably, 4µ8C treatment was initiated during a relatively early disease window, consistent with our transcriptomic observation that endothelial IRE1α activation emerges before overt end‐stage vascular destruction. This timing may be particularly relevant for future translational efforts, as therapies targeting endothelial stress responses may be most effective before irreversible vessel wall degeneration and hemorrhagic complications have become established.

The reduction in vascular Aβ40 burden after 4µ8C treatment raises an important mechanistic question, because IRE1α activation is likely downstream of Aβ40‐induced endothelial stress. Our additional APP analyses showed that 4µ8C did not significantly reduce APP mRNA or APP protein expression in APP23 mice, suggesting that this effect is unlikely to be primarily driven by decreased APP expression. A plausible explanation is that preservation of endothelial integrity may indirectly influence vascular Aβ40 accumulation [46]. Brain Aβ clearance is closely linked to vascular and perivascular routes, including BBB‐associated transport and intramural periarterial drainage [47, 48]. Disruption of endothelial junctions and vascular wall integrity may compromise these clearance pathways and promote local vascular retention of Aβ40 [46, 49, 50]. Therefore, by attenuating endothelial IRE1α signaling and preserving BBB integrity, 4µ8C may reduce vascular Aβ40 trapping or facilitate perivascular clearance. This interpretation remains provisional, and future studies directly assessing APP processing, Aβ production, and perivascular drainage function are needed.

Several limitations of the present study should also be acknowledged. First, although APP23 mice recapitulate important features of progressive amyloid deposition and vascular involvement, no single mouse model fully captures the complexity and heterogeneity of human sporadic CAA. Second, our mechanistic experiments were mainly performed in hCMEC/D3 cells, which provide a tractable endothelial system but do not fully reproduce the multicellular architecture of the neurovascular unit. Future studies incorporating primary endothelial cells, co‐culture systems, organoids, or in vivo endothelial‐specific genetic manipulations will be important to refine the cell‐autonomous and non‐cell‐autonomous contributions of IRE1α signaling. Third, although our data support a causal role for the IRE1α–TRAF2–JNK axis in endothelial apoptosis, the broader relationship among IRE1 RNase activity, inflammatory signaling, and vascular amyloid clearance remains to be defined [51]. Fourth, the human validation cohort was necessarily limited by surgical tissue availability and reflects advanced hemorrhagic disease; therefore, whether the same endothelial stress program is already present in earlier, non‐hemorrhagic stages of human CAA warrant further investigation.

In addition, the apparent decline of ER stress‐related scores at the latest disease stage deserves careful interpretation. Rather than indicating stress resolution, this pattern may reflect progressive endothelial depletion, phenotypic exhaustion, or selection for surviving cells with altered transcriptional capacity. In this regard, reduced late‐stage transcriptomic ER stress signals may paradoxically be compatible with more severe cumulative vascular injury. Longitudinal studies combining lineage‐aware transcriptomics, quantitative vascular pathology, and functional BBB assessment will be necessary to determine how endothelial stress trajectories relate to cell survival, compensation, and eventual barrier collapse over time.

In conclusion, this study identifies persistent endothelial ER stress as a central driver of CAA‐associated cerebrovascular injury and reveals the IRE1α–TRAF2 axis as a key molecular executor linking fibrillar Aβ40 to endothelial apoptosis and blood‐brain barrier breakdown. By integrating transcriptomic, cellular, pharmacological, and human pathological evidence, we provide a mechanistic framework in which endothelial vulnerability is positioned at the center of CAA progression. These findings not only deepen our understanding of how vascular amyloid translates into cerebrovascular dysfunction, but also nominate IRE1α‐directed intervention as a promising strategy for modifying the vascular course of CAA.

## 4 Experimental Section

### 4.1 Single‐Cell and Single‐Nucleus RNA Sequencing

#### 4.1.1 Tissue Dissociation and Cell Isolation

APP23 transgenic mice and age‐matched C57BL/6J wild‐type controls were used in this study. At each time point (3, 7, 12, and 15 months), cerebral cortices from five APP23 mice and five age‐matched WT mice were collected separately. For each genotype, cortical tissues from the five mice were pooled prior to tissue dissociation, fractionation, library preparation, and sequencing (Table S2). Following transcardial perfusion with ice‐cold PBS (HyClone, SH30256.01), pooled cerebral cortices were rapidly dissected on ice and minced into approximately 1 mm^3^ fragments using microsurgical scissors. Each pooled cortical sample was divided into two portions: approximately one‐third was reserved for snRNA‐seq nuclear isolation (N fraction), and the remaining two‐thirds were used for scRNA‐seq of cerebrovascular and glial cell populations.

For cerebrovascular cell isolation (E fraction), the two‐thirds cortical portion was transferred onto a 100 µm cell strainer and mechanically dissociated by gentle grinding. Material retained on the strainer was collected as the cerebrovascular‐enriched fraction. For glial cell isolation (G fraction), the flow‐through collected beneath the 100 µm strainer was retained. Both E and G fractions were subsequently dissociated using the Adult Brain Dissociation Kit (Miltenyi Biotec, 130‐107‐677) according to the manufacturer's instructions. When homogenate viscosity was elevated, DNase I (Sigma, 9003‐98‐9) was added to reduce DNA contamination. Following dissociation, erythrocytes were removed using red blood cell lysis buffer (Solarbio, R1010), and debris and dead cell removal was performed as needed using the Dead Cell Removal Kit (Miltenyi Biotec, 130‐109‐398/130‐090‐101). Cell viability and concentration were assessed using a Fluorescence Cell Analyzer (Countstar Rigel S2) with AO/PI reagent. Cells were washed twice in RPMI 1640 (Gibco, 11875119) and resuspended at 1 × 10^6^ cells/mL in RPMI 1640 supplemented with 2% FBS (Gibco, 10100147C) prior to library preparation.

#### 4.1.2 Nuclear Isolation

The one‐third cortical fraction reserved for snRNA‐seq (N fraction) was isolated using the Nucleus Isolation Kit (SHBIO, 52009–10), with RNase inhibitor (Sigma, 3335399001) added to all reagents prior to use to preserve RNA integrity. Tissue fragments were transferred into lysis buffer, mixed, and lysed on ice for 3 min. The lysate was filtered through a 40 µm cell strainer (Sigma, BAH136800040) to remove tissue debris. Nuclear concentration and viability were assessed using a Fluorescence Cell Analyzer (Countstar Rigel S2) with AO/PI reagent. Nuclear integrity was further confirmed by trypan blue staining (0.4%; Sangon Biotech, E607320‐0001) and visual inspection under a 40× microscope (Jiangnan Novel Optics, XD‐202). Only samples exhibiting intact nuclear envelopes and minimal impurities were proceeded to library preparation.

#### 4.1.3 Library Construction and Sequencing

scRNA‐seq libraries from E and G fractions and snRNA‐seq libraries from the N fraction were all prepared using the SeekOne DD Single Cell 3′ Library Preparation Kit (SeekGene, K00202), which employs droplet‐based microfluidic partitioning to capture individual cells or nuclei with unique molecular barcodes. Briefly, cells or nuclei were mixed with reverse transcription reagent and loaded onto a SeekOne DD Chip S3. Barcoded Hydrogel Beads (BHBs) and partitioning oil were dispensed into the corresponding wells to generate emulsion droplets, within which reverse transcription was performed at 42°C for 90 min and terminated at 85°C for 5 min. cDNA was purified from broken droplets and amplified by PCR, followed by fragmentation, end repair, A‐tailing, and sequencing adapter ligation. Indexed PCR was performed to selectively amplify the 3′ poly‐A fraction of expressed transcripts, incorporating cell barcodes and Unique Molecular Identifiers (UMIs) to enable single‐cell quantification and removal of PCR duplicates. Final libraries were purified using VAHTS DNA Clean Beads (Vazyme, N411‐01) and quality‐assessed using a Qubit fluorometer (Thermo Fisher Scientific, Q33226) and Bio‐Fragment Analyzer (Bioptic, Qsep400). All libraries were sequenced on an Illumina NovaSeq 6000 platform with paired‐end 150 bp read length.

### 4.2 Single‐Cell and Single‐Nucleus RNA Sequencing Data Processing and Cell Type Annotation

#### 4.2.1 Cell Quality Filtering

Raw gene expression matrices were generated by aligning sequencing reads to the mouse reference genome (mm10) using Cell Ranger (v7.1.0, 10x Genomics). Matrices were imported into R (v4.4.2) and processed using the Seurat package [52]. For each sample, cells were retained if they satisfied all of the following criteria: (1) the number of detected genes (nFeature_RNA) was between 500 and 6000; (2) the percentage of mitochondrial gene counts (percent_MT) was below 15%; and (3) the percentage of hemoglobin gene counts (percent_HB) was below 5%. These thresholds were applied to remove low‐quality cells, empty droplets, and potential erythrocyte contaminants. Of the 359 870 total barcodes detected, 313 718 cells passed initial quality filtering.

#### 4.2.2 Doublet Removal

Putative doublets were identified and removed using DoubletFinder (v2.0.3) applied independently to each sample prior to merging [53]. The optimal neighborhood proportion parameter (pK) was determined empirically for each sample via parameter sweep (paramSweep). DoubletFinder was run with the following parameters: pN = 0.25, nExp = 0.05 (corresponding to an expected doublet rate of 5%), PCs = 1:30, and n.neighbors = 30. Only singlets were retained. Following doublet removal, manual exclusion of clusters co‐expressing lineage‐incompatible marker combinations was performed to eliminate residual low‐quality populations. After all quality control steps, 288 496 high‐quality cells were retained for downstream analysis.

#### 4.2.3 Dimensionality Reduction, Clustering, and Batch Correction

Following quality control and doublet removal, the 288 496 high‐quality cells were re‐embedded for downstream analysis. Highly variable genes were identified using the “vst” method (nfeatures = 2000), and principal component analysis (PCA) was performed on the scaled expression matrix. To correct for technical variation across experimental batches, Harmony (v1.2.0) [54] was applied to the PCA embeddings with the following parameters: theta = 3, lambda = 0.1, max.iter.harmony = 20, and group.by.vars = “batch”. Unsupervised cell clustering was performed by constructing a shared nearest neighbor (SNN) graph on the first 30 dimensions of the Harmony‐corrected embeddings, followed by Louvain community detection at multiple resolutions; the final resolution was selected based on biological interpretability and marker gene consistency. Uniform Manifold Approximation and Projection (UMAP) was subsequently applied to the same 30‐dimensional Harmony‐corrected embeddings (dims = 1:30, all other parameters set to default) for 2D visualization of cell populations.

#### 4.2.4 Cell Type Annotation

Cell clusters were annotated manually based on the expression of canonical lineage marker genes. Cluster identity was assigned by examining the expression of established cell‐type‐specific markers across major brain vascular and parenchymal cell populations, including endothelial cells, smooth muscle cells, pericytes, astrocytes, microglia, oligodendrocytes, and neurons. Marker gene expression was visualized using two complementary approaches: kernel density estimation plots generated with the Plot_Density_Custom function from the scCustomize (v3.2.4) R package to display the continuous distribution of marker expression across the UMAP embedding (Figure S3), and dot plots generated using the DotPlot function in Seurat to summarize the average expression and detection rate of canonical markers across clusters (Figure 1D). Clusters exhibiting ambiguous or mixed marker expression were further examined and, where necessary, excluded as described above.

### 4.3 Differential Gene Expression and Functional Enrichment Analysis

#### 4.3.1 Differential Gene Expression Analysis

For each annotated cell type at each time point, differential gene expression analysis was performed by comparing APP23 cells with age‐matched WT cells. To avoid potential modality‐related confounding, each cell type was analyzed using either scRNA‐seq‐derived cells from the E/G fractions or snRNA‐seq‐derived cells from the N fraction, selected according to which sequencing modality provided better cell recovery and time point/genotype coverage for that cell type; the sample source used for each cell type is summarized in Table S3. Differentially expressed genes (DEGs) were identified using the FindMarkers function in Seurat (v5.0) with default parameters (Wilcoxon rank‐sum test). Genes were considered statistically significant if they met both of the following criteria: adjusted *p* value < 0.05 (Bonferroni correction) and |log_2_ fold change| > 0.25. Only genes detected in a minimum proportion of cells within the compared groups (min.pct = 0.1, default) were tested.

#### 4.3.2 Visualization of Differential Gene Expression

The distribution of significant upregulated and downregulated DEGs across cell types and disease stages was visualized using the ComplexHeatmap package (v2.26.1) in R. Upregulated DEGs were defined as genes with log_2_ fold change > 0.25 and adjusted *p* < 0.05 in APP23 mice relative to age‐matched WT controls, whereas downregulated DEGs were defined as genes with log_2_ fold change < −0.25 and adjusted *p* value < 0.05. For Figure 1G, H, the upper panels display DEGs shared across multiple cell type/time‐point comparisons, whereas the lower panels display DEGs specific to individual cell type/time‐point comparisons. Figure 2A summarizes the number of upregulated and downregulated DEGs across major cell types.

#### 4.3.3 Gene Ontology Enrichment Analysis

To characterize the biological functions of cell‐type‐ and stage‐specific DEGs, GO enrichment analysis was performed using the clusterProfiler package (v4.18.4) in R. For each cell type, the significant DEGs (adjusted *p*< 0.05, |log_2_FC| > 0.25) were used as input, with the full expressed gene set as background. Enrichment was assessed across all three GO categories: Biological Process (BP), Molecular Function (MF), and Cellular Component (CC). Terms were considered significantly enriched at an adjusted *p* value threshold of < 0.05 (Benjamini–Hochberg correction). Results were visualized using the enrichplot package.

### 4.4 Cell Type Proportion Analysis

#### 4.4.1 Single‐Cell Proportion Analysis

To quantify cell type composition across samples, the proportion of each cell type was calculated by dividing the number of cells assigned to each cell type by the total number of cells within the corresponding sample. Proportional differences between APP23 and age‐matched wild‐type C57BL/6J mice across time points were visualized as stacked bar plots.

#### 4.4.2 Bulk RNA‐Seq Deconvolution

To validate cell type proportion estimates in an independent bulk RNA‐seq dataset derived from cortical tissue, cellular deconvolution was performed using the MuSiC package (v1.0.0) [55]. A cell‐type‐specific reference signature matrix was constructed from the annotated N‐fraction single‐nucleus RNA‐seq data generated in this study, including APP23 and WT samples from all four time points. MuSiC was applied to bulk RNA‐seq expression profiles to estimate the relative abundance of each cell type in each sample. Between‐group differences in deconvolved cell type proportions between APP23 and wild‐type mice were assessed using Welch's *t*‐test, with statistical significance defined as *p* < 0.05. Results were visualized as bar plots with individual data points.

### 4.5 Pseudotime Trajectory Analysis

All pseudotime analyses were performed using scRNA‐seq‐derived endothelial cells from the vessel‐enriched (E) and glia‐enriched (G) fractions.

#### 4.5.1 Ordering Gene Selection

To reconstruct the transcriptional trajectory of endothelial cells during CAA progression, pseudotime analysis was performed using Monocle 2 (v2.38.0) [56]. In preliminary analyses, ordering genes selected from unsupervised highly variable genes or endothelial subcluster‐associated DEGs mainly reconstructed arterial–capillary–venous anatomical zonation rather than disease‐stage progression. Therefore, to focus the trajectory on CAA‐associated endothelial remodeling, ordering genes were selected based on disease‐associated differential expression. For each of the four time points examined, DEGs between APP23 and age‐matched WT endothelial cells were identified using the criteria described above (adjusted *p* < 0.05, |log_2_FC| > 0.25). The union of endothelial DEGs across the four age‐matched APP23‐vs‐WT comparisons was retained as the candidate ordering gene set. When a gene was detected as differentially expressed at more than one time point, the smallest adjusted *p* value across comparisons was used for ranking. The top 1500 genes ranked by adjusted *p* value were selected as the final ordering gene set for trajectory construction. This strategy was designed to enrich ordering genes for disease‐associated transcriptional perturbations while reducing the influence of baseline endothelial zonation and age‐related transcriptional changes.

#### 4.5.2 Trajectory Construction

To model the transcriptional dynamics of endothelial cells during CAA progression, trajectory analysis was restricted to endothelial cells from APP23 mice across all four time points examined. To mitigate the potential influence of unequal cell numbers across time points on trajectory construction and to facilitate visualization of the temporal distribution of cells along the inferred trajectory, 1500 endothelial cells were randomly downsampled from each of the four time point samples prior to analysis (random seed = 123). The selected ordering genes were used to reduce dimensionality via discriminative dimensionality reduction with trees (DDRTree), as implemented in Monocle 2. Cells were ordered along the inferred pseudotime trajectory using the orderCells function with default parameters. The root state of the trajectory was manually defined as the state predominantly occupied by 3‐month‐old APP23 endothelial cells, representing the earliest disease stage prior to substantial amyloid accumulation. All remaining parameters were set to Monocle 2 defaults.

#### 4.5.3 Pseudotime‐Dependent Gene Clustering and Functional Enrichment

Genes exhibiting significant variation along the pseudotime trajectory were identified using the differentialGeneTest function in Monocle 2 with default parameters, and genes with an adjusted *p* < 0.05 were considered pseudotime‐dependent. These genes were then grouped into co‐expression modules by unsupervised hierarchical clustering of their pseudotime‐dependent expression profiles. GO enrichment analysis of each gene cluster was performed using clusterProfiler as described above. Enriched GO terms with an adjusted *p* < 0.05 were considered statistically significant. For concise annotation and visualization of the major biological programs represented by each cluster, representative enriched terms passing a more stringent threshold of adjusted *p* value < 0.01 were selected for display.

### 4.6 Gene Set Scoring Analysis

#### 4.6.1 Gene Set Collection

Gene sets representing biological pathways and processes of interest were retrieved from the Molecular Signatures Database (MSigDB) using the msigdbr R package. Gene sets were drawn from three collections: Gene Ontology (GO) gene sets (C5 collection, encompassing Biological Process, Molecular Function, and Cellular Component sub‐ontologies), KEGG pathway gene sets (C2 collection), and Hallmark gene sets (H collection).

#### 4.6.2 Gene Set Scoring

Cell‐level gene set activity was quantified using two complementary scoring algorithms. First, AddModuleScore as implemented in Seurat (v5.0) was applied, which calculates the average expression of genes within each gene set relative to a randomly sampled background gene set of equal size, thereby controlling for differences in sequencing depth and gene detection rate. Second, UCell (v2.14.0) was applied as an independent scoring method, which computes per‐cell gene set enrichment scores based on the Mann–Whitney *U* statistic applied to gene expression rankings [57], providing a robust and sample‐size‐independent measure of gene set activity. Both scoring methods were applied to all cells independently, and the resulting scores were stored as cell‐level metadata. For visualization, AddModuleScore‐derived scores and UCell scores are displayed on the *y*‐axis and explicitly labeled accordingly to distinguish between the two methods.

### 4.7 Stratification of Endothelial Cells by ER Stress Activity and Pathway Analysis

All analyses in this section were performed using scRNA‐seq‐derived APP23 endothelial cells from the vessel‐enriched (E) and glia‐enriched (G) fractions.

#### 4.7.1 Cell Stratification by ER Stress Score

To investigate transcriptional programs associated with differential ER stress activity in endothelial cells, APP23 endothelial cells were stratified into ER stress‐high and ER stress‐low subpopulations based on their UCell‐derived ER stress gene set scores. Cells were ranked by score and assigned to the high or low group using the upper and lower quartiles as thresholds, respectively. Intermediate‐scoring cells were excluded from downstream comparisons to maximize contrast between groups.

#### 4.7.2 Differential Expression and Pathway Analysis between ER Stress Subpopulations

DEGs between ER stress‐high and ER stress‐low endothelial cells were identified using the FindMarkers function in Seurat (v5.0) with default parameters (Wilcoxon rank‐sum test, adjusted *p* < 0.05, |log_2_FC| > 0.25). GO enrichment analysis of the resulting DEGs was performed using clusterProfiler as described above, with significantly enriched terms (adjusted *p* < 0.05) visualized as bar plots to highlight ER stress‐associated biological processes.

### 4.8 Cell‐Cell Communication Analysis

All CellChat analyses were performed using scRNA‐seq‐derived cells from the vessel‐enriched (E) and glia‐enriched flow‐through (G) fractions. The N fraction was not included in this analysis.

#### 4.8.1 CellChat Object Construction and Preprocessing

To systematically investigate intercellular communication networks, cell–cell communication analysis was performed using CellChat (v2.1.2) with the mouse ligand‐receptor interaction database (CellChatDB.mouse) [58]. A CellChat object was constructed separately for each experimental group (APP23 and wild‐type mice at each time point) from the normalized single‐cell expression matrix and corresponding cell type annotations. Gene expression was averaged within each cell type using the computeCommunProb function, and communication probabilities were inferred based on the law of mass action, integrating ligand and receptor expression levels with cofactor and antagonist information. Signaling pathways were computed by aggregating communication probabilities across all ligand‐receptor pairs belonging to the same pathway using computeCommunProbPathway. All three categories of signaling interactions were included in the analysis: secreted signaling, extracellular matrix (ECM)‐receptor interactions, and cell–cell contact signaling.

#### 4.8.2 Multi‐Group Comparison

CellChat objects from APP23 and wild‐type groups across all time points were merged using the mergeCellChat function to enable systematic cross‐condition comparison. Overall changes in intercellular communication were assessed by comparing the total number of interactions and cumulative interaction strength between groups. Differential interaction analysis was performed to identify signaling pathways that were significantly up‐ or down‐regulated in APP23 relative to wild‐type mice, visualized as bubble plots and chord diagrams to illustrate the directionality and magnitude of communication changes across all cell type pairs.

#### 4.8.3 Endothelial Cell‐Focused Ligand‐Receptor Analysis

To characterize intercellular signals relevant to endothelial biology in CAA, ligand‐receptor pair analysis was focused on two functional categories: (1) tight junction‐associated signaling pairs mediating endothelial barrier integrity, and (2) proliferation‐regulatory ligand‐receptor pairs. Significant ligand‐receptor interactions involving endothelial cells as sender and receiver populations were extracted using default CellChat parameters and visualized as dot plots, with dot size representing communication probability and color indicating statistical significance. Ligand‐receptor pairs for which the inferred communication probability fell below the significance threshold in a given group were considered absent and are displayed as empty positions in the dot plot, indicating a loss of that specific intercellular interaction.

### 4.9 Virtual Knockout Analysis

Virtual knockout (KO) of Ern1 was performed using the scTenifoldKnk R package (v1.0.3), which enables in silico gene perturbation experiments from single‐cell RNA sequencing data without requiring matched experimental KO samples. Vascular endothelial cells from 12‐month‐old APP23 mice were extracted from the scRNA‐seq‐derived E and G fractions and used as input. Briefly, a single‐cell gene regulatory network (scGRN) was constructed from the normalized expression matrix of APP23 endothelial cells using principal component regression and low‐rank tensor decomposition. The target gene Ern1 was then virtually knocked out by setting all of its outgoing edges in the scGRN adjacency matrix to zero, and a second network was reconstructed from the perturbed matrix. Manifold alignment was subsequently applied to quantify the transcriptional perturbation of each gene as the result of Ern1 removal. Prior to network construction, genes expressed in fewer than 5% of cells were excluded, and the top 5000 genes ranked by mean normalized expression were retained; Ern1 was retained regardless of its rank. The analysis was run in “target” mode, in which the scGRN is constructed from the APP23 condition. For each gene, a *Z*‐score and fold change were computed from the manifold alignment distances, and statistical significance was assessed by chi‐squared test with Benjamini–Hochberg correction. Genes with FDR < 0.10 were considered significantly perturbed. All analyses were performed in R (v4.4.2) using parallel processing.

#### 4.9.1 Gene Set Enrichment Analysis

Gene Set Enrichment Analysis (GSEA) was performed on the full ranked gene list derived from the scTenifoldKnk output using the clusterProfiler R package (v4.14.0). Genes were ranked in decreasing order by their *Z*‐scores. Gene symbols were converted to Entrez IDs using the bitr function against the org.Mm.eg.db annotation database (v3.20.0). Gene sets for GO Biological Processes were retrieved from the MSigDB mouse gene set collection via the msigdbr R package (v25.1.1). GSEA was performed with a minimum gene set size of 10, a maximum of 500, and 1000 permutations with a fixed random seed. Normalized Enrichment Scores (NES) and false discovery rates (FDR) were computed using the standard GSEA algorithm. Pathways with FDR < 0.05 were considered significant. Enrichment plots were generated using the enrichplot R package (v1.26.1).

#### 4.9.2 Pathway and Process Enrichment Network Analysis

Pathway and process enrichment analysis of the significantly perturbed genes (*p* < 0.05, FDR < 0.10) identified by Ern1 virtual knockout was performed across multiple ontology sources including GO Biological Processes, GO Molecular Functions, KEGG Pathway, Reactome Gene Sets, Hallmark Gene Sets, Canonical Pathways, BioCarta Gene Sets, WikiPathways, and PANTHER Pathway. Terms with a *q*‐value < 0.01, a minimum count of 3, and an enrichment factor > 1.5 were retained and grouped into clusters based on membership similarity using kappa scores; sub‐trees with a similarity > 0.3 were considered a cluster, and the most statistically significant term within each cluster was used as the cluster label. The enrichment network was visualized in Cytoscape, where each node represents an enriched term (*q* < 0.01) and edges connect terms with a membership similarity > 0.3. Nodes are colored by cluster identity.

### 4.10 Patients

Human brain tissue samples were obtained from 32 patients with intracerebral hemorrhage, including 3 patients with CAA‐related intracerebral hemorrhage and 29 patients with hypertensive intracerebral hemorrhage, which served as non‐CAA hemorrhagic controls. The CAA‐related intracerebral hemorrhage group included 3 female patients, with a mean age of 72.7 ± 4.9 years. In the hypertensive intracerebral hemorrhage control group, 21 were male and 8 were female, with a mean age of 60.8 ± 12.2 years. The diagnosis of CAA‐related ICH was based on clinical radiological evaluations [35, 59] and strictly confirmed by postoperative pathological evidence of vascular Aβ40 deposition. This study was performed in accordance with the guidelines of the Declaration of Helsinki. All the participants or their legal proxies provided written informed consent before participating in the study. This study was approved by the Ethics Committee of the First Affiliated Hospital of Zhengzhou University (2025‐KY‐0395‐001).

### 4.11 Experimental Animals

Heterozygous APP23 transgenic mice (expressing the human APP751 isoform carrying the familial Swedish double mutation KM670/671NL under the control of the murine Thy1 promoter) [60] purchased from Jackson Laboratories and their age‐matched WT littermates on a C57BL/6 background were utilized in this study. Genotyping was routinely performed using genomic DNA extracted from tail snips via polymerase chain reaction (PCR). All animal experiments were conducted in accordance with the ARRIVE guidelines and approved by the Animal Ethics Committee of the First Affiliated Hospital of Zhengzhou University (2020‐KY‐0067‐001). All animals were maintained at a constant temperature (21°C±1°C) under 12‐h light/dark cycle and had free access to water and standard chow.

To evaluate the therapeutic efficacy of 4µ8C, seven‐month‐old APP23 mice and WT littermates were randomly allocated into the following three experimental groups (n = 7 mice per group): WT + Vehicle, APP23 + Vehicle, and APP23 + 4µ8C. The IRE1α inhibitor, 4µ8C (Selleck, S7272), was dissolved in vehicle containing 5% DMSO. Mice in the intervention group received daily intraperitoneal (i.p.) injections of 4µ8C at a dosage of 10 mg/kg, while the vehicle groups received equivalent volumes of the solvent. The intervention was administered continuously for 8 weeks. All mice were subjected to terminal procedures and tissue collection at exactly 9 months of age. Researchers involved in pathological quantifications and behavioral assessments were blinded to the group allocations.

To assess BBB integrity in vivo, 200 µl of sulfo‐NHS‐biotin (A8001, ApexBio, 4 mg per mouse) was retro‐orbitally injected into the mice (n = 3 mice per group) and circulated for 5 min to identify disrupted vessels in the brain. Following circulation, mice were euthanized and transcardially perfused with ice‐cold PBS to remove intravascular tracer, followed by perfusion with 4% paraformaldehyde (PFA) for tissue fixation. The fixed brains were cut into 80‐µm coronal sections on a vibratome (VT 1200 S, Leica).

#### 4.11.1 Morris Water Maze Test

The Morris water maze test was performed to assess spatial learning and memory after 4µ8C intervention. Mice from the WT + Vehicle, APP23 + Vehicle, and APP23 + 4µ8C groups were tested, with seven mice per group. The test was conducted in a circular water tank filled with opaque water maintained at 22°C–24°C. During the acquisition training phase, mice were trained to locate a hidden platform submerged below the water surface. Each mouse received four trials per day for five consecutive days. If a mouse failed to find the platform within 60 s, it was gently guided to the platform and allowed to remain there for 15 s. Escape latency was recorded as an index of spatial learning. Twenty‐four hours after the final training session, the platform was removed, and a probe trial was performed to evaluate spatial memory retention. The time spent in the target quadrant was recorded. Behavioral trajectories and parameters were recorded using an automated video tracking system.

### 4.12 Cell Cultures

Human cerebral microvascular endothelial cell line hCMEC/D3 cells obtained from Pricella (Wuhan, China, cat: CL‐0843) were cultured in Endothelial Cell Medium (ECM, Sciencell research laboratories, Cat. 1001), which was supplemented with 5% FBS, 1% endothelial cell growth supplement (ECGS), and 1% Penicillin‐Streptomycin (PS). The cells were maintained at 37°C in a humidified incubator with 5% CO_2_. The cells were routinely maintained within 20 passages to minimize phenotypic drift associated with long‐term culture. Mycoplasma contamination was monitored on a monthly basis using the MycoAlert Mycoplasma Detection Kit (Lonza, Switzerland), and all tests were consistently negative.

hCMEC/D3 cells were seeded and cultured until they reached approximately 70%–80% confluence. For Aβ40 PFF stimulation experiments, cells were treated with Aβ40 PFFs at the indicated concentrations for 24 h before protein extraction, immunofluorescence staining, flow cytometry, or co‐immunoprecipitation analysis. For IRE1α inhibition experiments, hCMEC/D3 cells were concurrently treated with Aβ40 PFFs and 4µ8C at 50 µm for 24 h before protein extraction. Control cells received an equal volume of vehicle.

The CELLImage Mini automated live‐cell imaging system (Chongqing Lianqing Ruiqi Co., Ltd., Chongqing, China) was surface‐sterilized with alcohol and placed inside a cell incubator. hCMEC/D3 cells were seeded into cell culture vessels at a density of 2 × 10^4^ cells/ cm^2^. Following Aβ40 PFFs treatment, the vessels were transferred to the observation area of the imaging system. A dynamic monitoring protocol was configured to capture cell images at an interval of 4 h per cycle, for a total of 6 cycles.

#### 4.12.1 Aβ40 PFFs Internalization and Colocalization Analysis

To visualize the cellular internalization of Aβ40 PFFs and assess their subcellular localization, hCMEC/D3 cells were seeded on glass coverslips and treated with Alexa Fluor 488‐labeled wild‐type Aβ40 PFFs (Aβ40 PFFs‐AF488) or AF488‐labeled Aβ40(L17A/F19A) mutant preparations at 10 µm for 24 h. For fluorescent labeling, Aβ40 PFFs or Aβ40(L17A/F19A) mutant preparations (100 µg) were labeled with Alexa Fluor 488 using an Alexa Fluor 488 labeling kit (Abcam, ab236553, Cambridge, UK) at a protein‐to‐dye ratio of 1:1 (w/w), according to the manufacturer's protocol. Briefly, the samples were incubated with Alexa Fluor 488 for 20 min at room temperature without agitation, as gentle static incubation was sufficient for efficient coupling. The labeling reaction was terminated by adding 20 µL quencher, followed by incubation for 5 min.

For endoplasmic reticulum visualization, live cells were incubated with ER‐Tracker Red (Beyotime, C1041S) according to the manufacturer's instructions prior to fixation. Subsequently, cells were fixed with 4% paraformaldehyde, permeabilized with 0.3% Triton X‐100, and blocked with 5% BSA. Cells were then incubated with an anti‐GRP78 primary antibody overnight at 4°C, followed by incubation with the appropriate fluorescent secondary antibody. F‐actin was stained using fluorescently labeled phalloidin (Proteintech, PF00001) to delineate cell boundaries, and nuclei were counterstained with DAPI.

All images were acquired using a Zeiss LSM 980 confocal microscope. To unequivocally confirm the intracellular localization of Aβ40 PFFs and rule out mere surface adhesion, confocal Z‐stack images were acquired across the cellular volume. Orthogonal projections (x‐z and y‐z planes) were then reconstructed from the Z‐stacks. Furthermore, to evaluate the spatial relationship between internalized Aβ40 PFFs, the ER network, and GRP78, colocalization analysis was performed. Line‐scan fluorescence intensity profiles were generated using ZEN software to quantify the spatial overlap and proximity of fluorescence peaks across selected regions of interest.

#### 4.12.2 siRNA Transfection

To knock down IRE1α expression, hCMEC/D3 cells were seeded in 6‐well plates and grown to 60%–70% confluence. Cells were transiently transfected with small interfering RNA (siRNA) targeting human ERN1 (IRE1α) or a non‐targeting negative control siRNA (si‐NC) using Lipofectamine RNAiMAX (Invitrogen, USA) according to the manufacturer's instructions. The specific target sequence for human IRE1α was 5'‐CCUCAACAUUACACUGCAA‐3'. After 24 h of transfection, the culture medium was replaced with fresh medium containing 10 µM Aβ40 PFFs. The cells were incubated with Aβ40 PFFs for an additional 24 h (total siRNA incubation time of 48 h) before being harvested for immunoblotting and flow cytometric apoptosis analysis.

### 4.13 Transmission Electron Microscopy

Human Aβ40 peptide and the fibrillization‐defective Aβ40(L17A/F19A) mutant peptide were purchased from GL Biochem (Shanghai) Ltd. (Shanghai, China). To prepare PFFs, peptide was dissolved in sterile PBS to a final concentration of 1 mg/mL and incubated at 37°C with continuous shaking at 1000 rpm for 7 days. The Aβ40 PFFs were diluted two‐fold and adsorbed to glow discharged 400 carbon‐coated copper grids for 2 min, quickly washed twice with Tris‐HCl (50 mm, pH 7.4), and floated upon two drops of 0.75% uranyl formatted for 30 s. The grids were allowed to dry before imaging under HT7800 TEM operating at 80 kV. The images were captured and digitized with an ER‐80 CCD (8 mega pixels).

The cells were cultured and treated with 10 µm PFFs on a Cell Desk polystyrene cover slip (Sumitomo Bakelite Co., Ltd.) at 25 000 cells per well, fixed with 2% formaldehyde and 2.5% glutaraldehyde in 0.1 m sodium‐phosphate buffer (pH 7.4) and washed three times for 5 min in the same buffer. The cells were post‐fixed for 1 h with 1% osmium tetroxide and 1% potassium ferrocyanide in 0.1 m sodium‐phosphate buffer (pH 7.4), dehydrated in a graded series of ethanol, and embedded in Epon812 (TAAB Co., Ltd.). Eighty nanometer ultra‐thin sections were stained with saturated uranyl acetate and lead citrate solution. Electron micrographs were obtained with a JEM‐1400plus transmission electron microscope (JEOL).

### 4.14 Immunostaining

For cell culture, hCMEC/D3 cells were seeded onto sterilized glass coverslips placed in 24‐well plates and treated with Aβ40 PFFs at a concentration of 10 µM for 24 h. After treatment, cells were fixed with 4% paraformaldehyde at room temperature for 15 min. The fixed cells were then washed three times with PBS and stored in PBS until further processing.

Both cell culture and tissue sections were permeabilized with 0.3% Triton X‐100 in PBS and blocked with 5% bovine serum albumin (BSA) in PBS. Sections were incubated with primary antibodies diluted in the blocking buffer overnight at 4°C. After primary antibody incubation, and washing three times with PBS, samples were incubated with fluorescently conjugated secondary antibodies (Alexa Fluor 488 and 594 conjugates, Abcam, 1:500) for 2 h at room temperature and washed three times with PBS before mounting with glass coverslips. Nuclei were stained with DAPI (4′,6‐diamidino‐2‐phenylindole). Confocal image stacks covering the entire cell volume were acquired using a Zeiss LSM 980 confocal microscope. Z‐stack images were collected where appropriate, and all imaging parameters were kept constant across groups for quantitative comparison.

### 4.15 Fluorescence Recovery After Photobleaching (FRAP) Analysis

A stable GFP‐GRP78‐overexpressing hCMEC/D3 cell line was generated by lentiviral transduction. Briefly, the full‐length human HSPA5/GRP78 coding sequence was cloned in‐frame with GFP into a lentiviral expression vector. The GRP78‐GFP lentivirus was constructed and packaged by GeneChem (Shanghai, China). hCMEC/D3 cells were infected with the GRP78‐GFP lentivirus according to the manufacturer's instructions, followed by puromycin selection to establish GRP78‐GFP‐overexpressing cells. For cellular FRAP analysis, GRP78‐GFP‐overexpressing hCMEC/D3 cells were seeded onto glass‐bottom dishes and cultured until they reached approximately 60%–70% confluence. Cells were then treated with thapsigargin (1 µm) or Aβ40 PFFs under the same conditions as described for the other Aβ40 PFFs stimulation experiments before live‐cell imaging.

FRAP experiments were performed using Zeiss LSM 980 confocal microscope equipped with a live‐cell incubation chamber maintained at 37°C and 5% CO_2_. GFP‐GRP78‐positive condensates were selected as regions of interest. After acquisition of pre‐bleach images, the selected region was photobleached using the 488‐nm laser at 80% laser power for 20 iterations, and fluorescence recovery was recorded at 1 s intervals. Fluorescence intensity within the bleached region was measured over time and corrected for background fluorescence and overall photobleaching during image acquisition. The normalized fluorescence recovery was calculated relative to the pre‐bleach fluorescence intensity and plotted as a function of time.

#### 4.15.1 Recombinant GRP78 Protein Purification and In Vitro LLPS Assay

Protein Expression and Purification: The full‐length human HSPA5 (GRP78) coding sequence was cloned into the pET28a‐6His‐tev expression vector. The recombinant construct was transformed into E. coli Rosetta (DE3) cells. Cells were grown in auto‐induction medium at 37°C for 3–4 h, followed by incubation at 25°C for 20 h. Cells were harvested by centrifugation and lysed by sonication in binding buffer (50 mm NaH_2_PO_4_, 300 mm NaCl, 10 mm imidazole, pH 8.0) supplemented with 1 mm PMSF. The lysate was cleared by centrifugation, and the supernatant was loaded onto a Ni‐NTA affinity column (HisTrap HP 5 mL, GE Healthcare). Following extensive washing, the bound proteins were eluted with a linear gradient of imidazole (up to 250 mm). For further purification, the GRP78‐containing fractions were desalted into 20 mm Tris‐HCl (pH 7.0) and subjected to anion exchange chromatography using a Capto Q ImpRes column. The bound proteins were eluted using a linear gradient of 1 m NaCl. Finally, the purified GRP78 protein was concentrated and buffer‐exchanged into 1× PBS (pH 7.4) using Amicon Ultra‐15 centrifugal filter units (30 kDa MWCO). Protein purity was assessed by SDS‐PAGE followed by Coomassie Brilliant Blue staining.

For the in vitro LLPS assays, purified GRP78 protein was diluted to a final concentration of 5 µm in a phase separation buffer comprising 20 mm Tris‐HCl, 150 mm NaCl, pH 7.4, 10% PEG‐8000. To evaluate the effect of Aβ40 on GRP78 phase separation, Aβ40 PFFs were added to the mixture at indicated concentrations and incubated at 37°C for 2 h. The protein mixtures were then deposited onto a glass‐bottom 384‐well microplate. Droplet formation and morphology were immediately visualized and captured using a Zeiss LSM 980 confocal microscope.

### 4.16 Apoptosis Detection by Flow cytometry (FCM)

Apoptosis was assessed using an Annexin V‐PE/PI staining kit (BioLegend, 640914). Cells were cultured in 6‐well plates and treated as described. After treatment, cells were harvested by trypsinization, washed with phosphate‐buffered saline (PBS), and resuspended in binding buffer. The cells were then stained with Annexin V‐APC and Propidium Iodide (PI) according to the manufacturer's protocol. Briefly, 5 µL of Annexin V‐PE and 5 µL of PI were added to the cells, followed by incubation for 15 min at room temperature in the dark. The samples were analyzed immediately using a BD LSRFortessa flow cytometer (BD Biosciences). The flow cytometry data were analyzed using FlowJo software.

The results were plotted on a 2D dot plot where the *x*‐axis represents Annexin V‐PE fluorescence intensity (Comp‐APC‐A::Annexin V), and the *y*‐axis represents PI fluorescence intensity (Comp‐PE‐A::PI). Quadrant analysis was used to categorize the populations into early apoptotic (Annexin V‐positive/PI‐negative), late apoptotic (Annexin V‐positive/PI‐positive), and viable cells (Annexin V‐negative/PI‐negative).

### 4.17 Western Blot Analysis

RIPA lysis buffer supplemented with protease and phosphatase inhibitors was added to the cells. The samples were incubated on ice for 30 min and centrifuged at 12 000 × g for 10 min at 4°C. The supernatants containing total proteins were collected, and protein concentrations were determined using a BCA protein assay according to the manufacturer's instructions. Equal amounts of protein samples, 10 µg per lane, were mixed with 5× loading buffer and boiled at 100°C for 10 min. Proteins were separated by sodium dodecyl sulfate‐polyacrylamide gel electrophoresis (SDS‐PAGE), together with a pre‐stained protein marker (26616, Thermo Fisher Scientific), and then transferred onto 0.2‐µm polyvinylidene fluoride membranes (ISEQ00010, Millipore, USA). The membranes were blocked with 5% skimmed milk in 1× Tris‐buffered saline with Tween 20 (TBST) for 1 h at room temperature, followed by incubation with primary antibodies diluted in 5% skimmed milk at 4°C overnight. After washing three times with TBST, the membranes were incubated with the corresponding secondary antibodies at a dilution of 1:10 000 for 1.5 h at room temperature. Finally, the membranes were washed three times with TBST and visualized using enhanced chemiluminescence reagent (39864S, Cell Signaling Technology). Protein bands were detected using a Bio‐Rad ChemiDoc Imaging System. The primary antibodies used in this study are listed in Table S1.

### 4.18 Co‐Immunoprecipitation (Co‐IP) Assay

hCMEC/D3 cells were treated with Aβ40 PFFs as described above and washed twice with ice‐cold PBS. Cells were lysed on ice in ice‐cold IP lysis buffer supplemented with protease and phosphatase inhibitor cocktails. The lysates were clarified by centrifugation at 12 000 × g for 15 min at 4°C, and protein concentrations were determined using a BCA protein assay. Equal amounts of protein from each sample were incubated with anti‐IRE1α antibody or control IgG overnight at 4°C with gentle rotation. Protein A/G magnetic beads were then added and incubated for 2 h at 4°C. After incubation, the beads were washed three times with IP lysis buffer, and bound proteins were eluted by boiling in SDS loading buffer. The immunoprecipitated proteins and corresponding input lysates were separated by SDS‐PAGE and analyzed by immunoblotting. The primary antibodies used in this study are listed in Table S1.

#### 4.18.1 Multiplex Immunohistochemical Staining (mIHC)

The collected tissue sections were dewaxed with conventional xylene and hydrated with gradient alcohol. mIHC of the tissue sections was performed using a seven‐color multiplex immunofluorescence staining kit (AFIHC027, AiFang Biologcal, China). According to the manufacturer's instructions, the antigens were exposed by microwave repair. The sections were incubated with a 3% hydrogen peroxide solution at room temperature for 15 min, and then dropped with 10% goat serum for blocking for 15 min. The primary antibody A was added and incubated overnight at 4°C, and then washed three times with PBST. Polymer‐HRP anti‐mouse/rabbit universal secondary antibody IgG (AFIHC001, AiFang Biologcal) was dropped, and the sections were incubated at room temperature for 30 min. Subsequently, the sections were washed with PBST, and the TYR fluorescent dye was dropped and reacted for 8 min, followed by three washes with PBST. Antibody A was removed by microwave treatment, and the sections were washed three times with PBST. After dropping goat serum for blocking, the primary antibody B was added, and the operation was repeated until all the antigens, which are listed in the Table S1, were completely visualized. DAPI staining solution was dropped, and the sections were incubated at room temperature in the dark for 10 min, followed by three washes with PBST. The sections were mounted with an anti‐fluorescence quenching mounting medium, and the mIHC slides were imaged using an eight‐channel fluorescence digital slide scanner (model AF‐KL‐20‐8, AiFang Biologcal, China).

### 4.19 Statistical Analysis

Statistical analyses were performed using GraphPad Prism 8 software. All data were tested for normality and variance. If significant, the student's *t*‐test was used to compare two groups or one‐way analysis of variance followed by Tukey's post‐hoc test was used to compare more than two groups. The data are presented as the means ± SEM. The sample size for each statistical analysis is indicated in the corresponding figure legend. Statistical significance was set at *P* <0.05.

## Author Contributions

Conceptualization: HZ and QL; Methodology: HZ and QL; Investigation: HZ and QL; Visualization: HZ and QL; Software: QL; Formal Analysis: QL; Validation: NZ, YY, YL, SD, JZ, HZ, CL, YZ, WZ, YS, TW, YL, HL and YG; Supervision: YX, ZX and HL; Writing – original draft: HZ and QL; Writing – review & editing: YX, ZX and HL.

## Funding

The authors’ work was supported by the National Natural Science Foundation of China (Grants 82301619, 32470159 & 82571514), the key scientific and technological breakthrough project in Henan province (Grant 232102311229) and the China Postdoctoral Science Foundation (Grant 2022M722875 & 2023T160600).

## Declarations‐Ethics approval and Consent to Participate

This study was performed in accordance with the guidelines of the Declaration of Helsinki. All the participants or their legal proxies provided written informed consent before participating in the study. All animal experiments were conducted in accordance with ARRIVE guidelines. All studies were approved by the Ethics Committee of the First Affiliated Hospital of Zhengzhou University (2025‐KY‐0395‐001 and 2020‐KY‐0067‐001).

## Conflicts of Interest

The authors declare no conflicts of interest.

## Supporting information




**Supporting File 1**: advs76761‐sup‐0001‐SuppMat.docx.

**Supporting File 2**: advs76761‐sup‐0002‐TableS2.xlsx.

**Supporting File 3**: advs76761‐sup‐0003‐TableS3.xlsx.

## Data Availability

The processed data supporting the findings of this study are available in the Supplementary Information. The raw sequencing data generated in this study are available from the corresponding authors upon reasonable request.
