# Supplementary material for: Aberrant GRP78 Phase Transition Sustains Endothelial IRE1α Signaling and Drives Blood–Brain Barrier Failure in Cerebral Amyloid Angiopathy
Source: Adv Sci (Weinh). 2026 Jul 24;13(58):e76761. doi: 10.1002/advs.76761 (PMC13398133; doi:10.1002/advs.76761)
Supplement: Supplementary file 1 [file ADVS-13-e76761-s001.docx]

Aberrant GRP78 Phase Transition Sustains Endothelial IRE1α Signaling and Drives Blood–Brain Barrier Failure in Cerebral Amyloid Angiopathy

*Honglin Zheng, Qiang Li, Haiyang Luo^*^, Yapei Yuan, Na Zhang, Yongting Lu, Suying Duan, Jieshi Zhong, Hang Zhang, Chenyang Liu, Yaochong Zhang, Wenzhuo Zhao, Yaxuan Song, Tuo Wang, Si Yan, Yapeng Li, Han Liu,* *Yuan Gao, Zongping Xia^*^, and Yuming Xu**^*^*

**Supplementary information**

**Figure S1- Figure S22**

**Supplementary Table S1**

**Figure S1: Quality control, filtering, and integration of the single-cell and single-nucleus transcriptomic dataset. Related to Figure 1.**

(A) UMAP visualization of the initial integrated dataset before stringent quality control, colored by unsupervised Seurat clusters.
(B–D) Scatter plots showing the relationships between nCount_RNA and nFeature_RNA (B), percent_MT (C), and percent_HB (D) across all cells and nuclei in the pre-filtered dataset. Correlation coefficients are indicated in each panel.
(E–H) Violin plots displaying the distributions of nCount_RNA (E), nFeature_RNA (F), percent_MT (G), and percent_HB (H) across individual samples after quality control filtering, labeled by genotype, age, and fraction type.
(I) UMAP visualization showing cells/nuclei retained (Included) or removed (Excluded) after quality-control filtering. Low-quality profiles were mainly located at cluster boundaries or in isolated peripheral regions.
(J–L) Scatter plots showing the relationships between nCount_RNA and nFeature_RNA (J), percent_mito (K), and percentHB (L) in the filtered dataset. Correlation coefficients are indicated in each panel.
(M–P) UMAP feature plots of nCount_RNA (M), nFeature_RNA (N), percent_MT (O), and percent_HB (P) across the filtered dataset.
(Q–S) UMAP visualizations of the filtered and integrated dataset colored by individual sample (Q), genotype/age group (R), and reclustered Seurat clusters (S).

**Figure S2: Doublet detection across single-cell and single-nucleus transcriptomic samples using DoubletFinder. Related to Figure 1.**

UMAP visualization of 24 samples after DoubletFinder classification, showing cells identified as doublets (red) versus singlets (blue). The samples are classified into three types: E (vascular-enriched single-cell sequencing, manually selected under a microscope), G (single-cell sequencing excluding vascular cells), and N (single-nucleus sequencing). Data are presented for each genotype (APP23 and WT) at four different time points (3, 7, 12, and 15 months).

**Figure S3: UMAP visualization of cell-type-specific marker expression across the integrated dataset. Related to Figure 1.**

UMAP feature plots showing the expression density of 36 cell-type-specific markers across the dataset. Each panel represents the expression of a different marker gene, with color intensity indicating the density of expression across cells. The color scale (shown on the right) represents the density of gene expression, ranging from low (black) to high (purple).

**Figure S4. Distribution of the human APP751 transgene and cell-type composition across** **fractionated samples. Related to Figure 1 & Figure 2.**
(A) UMAP feature plot showing the expression of the human APP751 transgene (hAPP751) across the integrated dataset.
(B) Violin plot showing hAPP751 expression levels across annotated cell types. Statistical significance was assessed by Kruskal–Wallis test.
(C–E) Stacked bar plots showing the relative cell-type composition across the following fractions: (C) glia-enriched fractions (G), (D) neuron-enriched fractions (N), and (E) vascular-enriched fractions (E) from APP23 and wild-type (WT) mice at 3, 7, 12, and 15 months.
(F–G) Bar graphs quantifying the relative cellular abundance of smooth muscle cells (SMCs) (F) and pericytes (G) estimated via deconvolution of bulk RNA-seq data using the MuSiC algorithm across the spatiotemporal continuum (3, 7, 12, and 15 months). Statistical significance was assessed using Welch’s t-test. Data are presented as mean ± SEM. **P* < 0.05.

**Figure S5. Stage-resolved transcriptional remodeling of endothelial cells and smooth muscle cells during CAA progression. Related to Figure 1.**
(A) Representative differentially expressed genes (DEGs) in endothelial cells and smooth muscle cells (SMCs) from APP23 mice relative to age-matched wild-type (WT) controls at 3, 7, 12, and 15 months. Upregulated genes are shown in red and downregulated genes in blue. Top 3 upregulated and downregulated DEGs for each cell type and disease stage are indicated.
(B–E) Functional enrichment analysis of DEGs in endothelial cells at 3 months (B), 7 months (C), 12 months (D), and 15 months (E).
(F–I) Functional enrichment analysis of DEGs in SMCs at 3 months (F), 7 months (G), 12 months (H), and 15 months (I).

**Figure S6. Comparison of UCell scores for endoplasmic reticulum stress- and tight junction-related gene signatures between APP23 and WT groups across ages. Related to Figure 2 & Figure 4.**

(A) UCell scores of the response to endoplasmic reticulum stress gene set in APP23 and WT groups at 3, 7, 12, and 15 months.
(B) UCell scores of the unfolded protein response gene set in APP23 and WT groups at 3, 7, 12, and 15 months.
(C) UCell scores of the KEGG_TIGHT_JUNCTION gene set in APP23 and WT groups at 3, 7, 12, and 15 months.
****, *P* < 0.0001 by Wilcoxon rank-sum test.

**Figure S7. Branch-specific analysis of unfolded protein response signaling in endothelial cells during CAA progression. Related to Figure 2.**

(A-F) Violin plots quantifying the activation status of the three classical Unfolded Protein Response (UPR) branches in endothelial cells using two independent scoring methodologies: AddModuleScore (left panels) and UCell Score (right panels). ***P* < 0.01, **** *P* < 0.0001, ns = not significant by Wilcoxon rank-sum test.
(G-H) Split violin plots illustrating the expression kinetics of representative core UPR genes, *Hspa5* (GRP78) (G) and *Xbp1* (H), in endothelial cells. Data are presented as median ± interquartile range (IQR) for boxplots. **** *P* < 0.0001 by Wilcoxon rank-sum test.

**Figure S8.** **Age-dependent activation of the IRE1α–XBP1 signaling axis in APP23 mice. Related to Figure 2.**
(A, B) Multiplex immunohistochemistry of brain sections from wild-type (WT) (A) and APP23 (B) mice at 3 and 7 months of age stained for CD31, p-IRE1, IRE1, XBP1-S, XBP1-U, and DAPI. WT mice showed low basal vascular staining of IRE1 pathway markers at both time points. Scale bars, 500 μm (whole-section view) and 100 μm (magnified views). (C, D) Quantification of relative p-IRE1α/IRE1α (C) and XBP1-S/XBP1-U (D) fluorescence intensity ratios in brain sections from 3-, 7-, and 12-month-old WT and APP23 mice. Ratios were normalized to the mean value of the 3-month-old WT group. Data are presented as mean ± SEM. Statistical significance was determined by two-way ANOVA followed by Tukey’s post hoc test. P values indicate genotype comparisons within each age group.

**Figure S9. Quantitative biochemical analysis of Aβ40-induced UPR activation and validation of recombinant GRP78 purity. Related to Figure 3.**

(A–E) Quantification of protein expression levels for GRP78/GAPDH (A), p-IRE1α/IRE1α (B), p-IRE1α/GAPDH (C), XBP1-S/XBP1-U (D), and XBP1-S/GAPDH (E) in hCMEC/D3 cells treated with increasing concentrations of Aβ40 preformed fibrils (PFFs) (0 to 20 μM). These quantitative data correspond to the representative immunoblots shown in Fig. 3A. Data are presented as mean ± SEM (n=3).
(F) Coomassie Brilliant Blue staining showing the purified GRP78 protein.

**Figure S10. Structural characterization of the fibrillization-defective Aβ40(L17A/F19A) mutant. Related to Figure 3.**
(A) Representative transmission electron microscopy images of WT Aβ40 and the Aβ40(L17A/F19A) mutant. Scale bars, 500 nm (upper panels) and 200 nm (lower panels).
(B) Representative atomic force microscopy images of WT Aβ40 and Aβ40(L17A/F19A). Scale bars, 600 nm.

**Figure S11. A fibrillization-defective Aβ40 mutant exhibits attenuated ER stress-inducing and junction-disrupting activity in hCMEC/D3 cells. Related to Figure 3 & Figure 4.**

(A) Representative immunoblots of GRP78, p-IRE1α, IRE1α, XBP1-S, XBP1-U, Occludin, JAM-A, ZO-1, and GAPDH in hCMEC/D3 cells treated with vehicle control, WT Aβ40 PFFs, or the fibrillization-defective mutant Aβ40(L17A/F19A) PFFs.
(B–I) Quantification of GRP78/GAPDH (B), p-IRE1α/GAPDH (C), p-IRE1α/IRE1α (D), XBP1-S/GAPDH (E), XBP1-S/XBP1-U (F), Occludin/GAPDH (G), JAM-A/GAPDH (H), and ZO-1/GAPDH (I) from the immunoblots shown in (A). Data are presented as mean ± SEM.
(J, L) Immunofluorescence staining of XBP1-S (J) and p-IRE1α (L) in hCMEC/D3 cells treated with vehicle control, WT Aβ40 PFFs, or Aβ40(L17A/F19A) PFFs. Nuclei were counterstained with DAPI. Scale bars, 20 μm.
(K, M) Quantification of relative fluorescence intensity of XBP1-S (K) and p-IRE1α (M) from (J) and (L), respectively. Data are presented as mean ± SEM.

**Figure S12. Internalized Aβ40 PFFs spatially encounter ER-associated GRP78 in endothelial cells. Related to Figure 3.**

(A) Representative confocal images of hCMEC/D3 cells treated with PBS or Aβ40 PFFs-AF488 and stained with phalloidin and DAPI. Aβ40 PFFs-AF488 signals were detected as puncta in endothelial cells after Aβ40 PFF treatment. Scale bar, 10 μm. (B) Orthogonal Z-stack reconstruction showing intracellular localization of Aβ40 PFFs-AF488 signals in hCMEC/D3 cells. Scale bar, 20 μm. (C) Representative confocal images of hCMEC/D3 cells treated with PBS, Aβ40 PFFs-AF488, or Aβ40 (L17A/F19A)-AF488 and stained with ER-Tracker and DAPI. Scale bar, 10 μm. (D) Line-scan fluorescence intensity profiles showing the spatial relationship among Aβ40 PFFs-AF488, ER-Tracker, and DAPI signals. (E) Representative confocal images showing Aβ40 PFFs-AF488, GRP78, phalloidin, and DAPI staining in hCMEC/D3 cells. Internalized Aβ40 PFFs-AF488 signals showed partial spatial association with GRP78-positive structures. Scale bar, 10 μm.

 **Figure S13. Global cell-cell communication networks across disease stages in WT and APP23 mice. Related to Figure 4.**

(A, B) Circle plots showing the global intercellular communication landscape among major annotated cell types in wild-type (WT) and APP23 mice at 3, 7, 12, and 15 months of age. (A) Interaction number and (B) interaction strength are shown for each group. Nodes represent cell types, and node size reflects the number of cells. Edges indicate inferred intercellular communication links, with edge number or thickness corresponding to the total number (A) or overall strength (B) of interactions between cell types.

**Figure S14. Stage-resolved outgoing and incoming interaction strength of major cell types in APP23 and WT mice. Related to Figure 4.**
Bubble plots showing the outgoing interaction strength (x-axis) and incoming interaction strength (y-axis) of major annotated cell types in APP23 and WT mice at 3, 7, 12, and 15 months of age. Each dot represents one cell type, and dot size reflects the number of interactions.

**Figure S15. Cell-cell communication probabilities across different time points in APP23 and WT mice. Related to Figure 4.**
The plot shows the communication probabilities (Commun_prob) of representative ligand-receptor pairs in APP23 and WT mice across different ages. Dot color intensity corresponds to the communication level. A red box highlights ligand-receptor pairs whose communication is lost during disease progression. The absence of a dot indicates no detectable communication.

**Figure S16. Early-stage multiplex immunohistochemical analysis of endothelial junction organization in WT and APP23 mice. Related to Figure 4.**
(A, B) mIHC of brain sections from wild-type (WT) (A) and APP23 (B) mice at 3 and 7 months of age stained for JAM-A, Occludin, Aβ, α-SMA, CD31, and DAPI. In WT mice, JAM-A and Occludin remained continuously distributed along the cerebrovascular wall at both 3 and 7 months. Arrows indicate sparse Aβ-positive signals in 7-month-old APP23 brain sections. Scale bars, 500 μm (whole-section view) and 100 μm (magnified views).

**Figure S17. Early-stage mIHC analysis of endothelial apoptosis and cell death markers in WT and APP23 mice. Related to Figure 5.**

(A, B) Multiplex immunohistochemistry of brain sections from wild-type (WT) (A) and APP23 (B) mice at 3 and 7 months of age stained for GSDMD-NT, p-RIPK1, Cleaved Caspase-3, NeuN, p-MLKL, and DAPI. Scale bars, 500 μm (whole-section view) and 100 μm (magnified views).

**Figure S18. Virtual knockout of *Ern1* in vascular endothelial cells from 12-month-old App23 mice implicates blood-brain barrier dysfunction, neuroinflammation, and JNK-mediated apoptosis in cerebral amyloid angiopathy.** **Related to Figure 5.**

(A) Volcano plot showing the transcriptional perturbation magnitude of all genes following virtual knockout of *Ern1* in vascular endothelial cells from App23 mice, as computed by scTenifoldKnk. The x-axis represents the log₂-transformed perturbation magnitude (fold change), and the y-axis represents the −log₁₀-transformed false discovery rate (FDR). The dashed horizontal line indicates the FDR = 0.10 significance threshold. Genes with FDR < 0.10 are highlighted in red. Ern1 (the perturbed gene) is indicated by a triangle marker. The top 15 most significantly perturbed genes are labeled.
(B) Bubble plot summarizing representative Gene Set Enrichment Analysis (GSEA) results for genes perturbed by *Ern1* virtual knockout, grouped into eight biologically relevant themes. The x-axis represents the Normalized Enrichment Score (NES); positive NES indicates pathway activation and negative NES indicates suppression. Bubble size reflects the −log₁₀(FDR). Pathways are colored by biological theme as indicated by the labels.
(C) GSEA enrichment plot for the gene ontology biological process term "Positive Regulation of JNK Cascade" (GO:0046330). The curve shows the running enrichment score across the gene list ranked by Z-score from the scTenifoldKnk analysis.
(D) Pathway and process enrichment network for the 46 significantly perturbed genes identified by Ern1 virtual knockout (*P* < 0.05, FDR < 0.10). Each node represents an enriched term (q < 0.01); nodes are colored by cluster identity. Edges connect terms with membership similarity > 0.3.

**Figure S19. Densitometric quantification of ER stress and apoptosis-related proteins in hCMEC/D3 cells treated with different concentrations of Aβ40 PFFs. Related to Figure 5E.**

(A–G) Relative protein expression levels of (A) p-IRE1α/IRE1α, (B) p-IRE1α/GAPDH, (C) TRAF2/GAPDH, (D) p-JNK/GAPDH, (E) Bcl-2/GAPDH, (F) Bax/GAPDH, and (G) cleaved Caspase-3/GAPDH. Data are presented as mean ± SEM from three independent biological replicates (n = 3). Statistical significance was determined by one-way ANOVA followed by Tukey’s post hoc test. Exact *P* values are indicated above the bars.

**Figure S20. Densitometric quantification of ER stress- and apoptosis-related proteins after IRE1α knockdown in hCMEC/D3 cells. Related to Figure 5G.**

(A–G) Relative protein expression levels of (A) p-IRE1α/IRE1α, (B) p-IRE1α/GAPDH, (C) TRAF2/GAPDH, (D) p-JNK/GAPDH, (E) Bcl-2/GAPDH, (F) Bax/GAPDH, and (G) cleaved Caspase-3/GAPDH in control, Aβ40 PFF-treated, si-IRE1α-treated, and si-IRE1α plus Aβ40 PFF-treated hCMEC/D3 cells. Data are presented as mean ± SEM from three independent biological replicates (n = 3). Statistical significance was determined by one-way ANOVA followed by Tukey’s post hoc test. Exact *P* values are indicated above the bars.

**Figure S21. 4μ8C treatment does not reduce APP expression.**

(A) Quantitative PCR analysis of APP mRNA expression in WT+Vehicle, APP23+Vehicle, and APP23+4μ8C mice. APP mRNA expression was significantly increased in APP23 mice compared with WT mice, whereas 4μ8C treatment did not significantly reduce APP mRNA expression in APP23 mice.

(B) Representative Western blot images showing APP protein expression in WT+Vehicle, APP23+Vehicle, and APP23+4μ8C mice. GAPDH was used as the loading control.

(C) Quantification of APP protein levels normalized to GAPDH. APP protein abundance was increased in APP23 mice compared with WT mice, whereas no significant reduction was observed after 4μ8C treatment. Data are presented as mean ± SEM. P values are indicated above the comparisons.

**Figure S22. Representative neuroimaging of patients with hypertensive intracerebral hemorrhage and CAA-related intracerebral hemorrhage. Related to Figure 7.**

(A–F) Representative neuroimaging of a patient with hypertensive intracerebral hemorrhage (ICH).
(A) Non-contrast CT showing an acute deep macroscopic hemorrhage in the left basal ganglia.
(B) Susceptibility-weighted imaging (SWI) displaying the corresponding deep hemorrhage. (C) SWI showing an absence of lobar microbleeds.
(D) SWI revealing multiple deep cerebral microbleeds (CMBs) in the basal ganglia and thalamus.
(E) T2-weighted imaging demonstrating enlarged perivascular spaces predominantly in the basal ganglia (BG-EPVS). (F) Fluid-attenuated inversion recovery (FLAIR) showing white matter hyperintensities (WMH).
(G–L) Representative neuroimaging of a patient with CAA-related intracerebral hemorrhage.
(G) Non-contrast CT showing an acute lobar hemorrhage in the right parieto-occipital region. (H) SWI displaying the corresponding macroscopic lobar hemorrhage.
(I) SWI revealing focal cortical superficial siderosis (cSS).
(J) SWI showing multiple, strictly lobar CMBs.
(K) T2-weighted imaging demonstrating enlarged perivascular spaces predominantly in the centrum semiovale (CSO-EPVS).
(L) FLAIR showing severe periventricular and deep WMH.

**Supplementary Table S1. The main primary antibodies used in this study.**

| **Primary antibodies** | **Company** | **Cat.** | **Dilution** |
| --- | --- | --- | --- |
| β-Amyloid | Biolegend | 9200-02 | IHC: 1:1000 |
| β-Amyloid 1-40 | Abcam | ab20068 | mIHC: 1:200 |
| IRE1 | Affinity | DF7709 | mIHC: 1:300  WB: 1:1000 |
| Phospho-IRE1 (Ser724) | Zenbio | 530878 | mIHC: 1:400  IF: 1:500  WB: 1:1000 |
| XBP1-U | Proteintech | 25997-1-AP | mIHC: 1:200  WB: 1:1000 |
| XBP1-S | Proteintech | 24868-1-AP | mIHC: 1:800  IF: 1:500  WB: 1:1000 |
| CD31 | AiFang biological | AFRM0001 | mIHC: 1:300 |
| JAM-A | Affinity | DF6373 | mIHC: 1:800 |
| Occludin | AiFang biological | AFRP0025 | mIHC: 1:300 |
| Occludin | Proteintech | 27260-1-AP | WB: 1:10000 |
| α-SMA | AiFang biological | AFMM0002 | mIHC: 1:500 |
| GRP78 | AiFang biological | AFRMO138 | mIHC: 1:400 |
| GRP78 | Selleck | F0221 | WB: 1:1000 |
| CHOP | AiFang biological | AFRM0253 | mIHC: 1:200 |
| ZO-1 | Proteintech | 21773-1-AP | WB: 1:10000 |
| GAPDH | Proteintech | 10494-1-AP | WB: 1:10000 |
| JAM-A | Proteintech | 84720-2-RR | WB: 1:10000 |
| Collagen IV | Affinity | AF0510 | IF: 1:200 |
| TRAF2 | Proteintech | 26846-1-AP | WB: 1:2000 |
| Phospho-JNK (Tyr185) | Proteintech | 80024-1-RR | WB: 1:2000 |
| BCL2 | Proteintech | 60178-1-Ig | WB: 1:2000 |
| BAX | Proteintech | 50599-2-Ig | WB: 1:20000 |
| Cleaved Caspase-3 (Asp175) | Cell Signaling Technology | 9661S | WB: 1:1000 |
| Cleaved-Caspase-3 | AiFang biological | [AFRP0036](http://afantibody.cn/product/show/26903.html#/experiment/_blank) | mIHC: 1:300 |
| Cleaved N-terminal GSDMD | MedChemExpress | HY-P86158 | mIHC: 1:300 |
| Phospho-MLKL (Ser345) | Cell Signaling Technology | 37333 | mIHC: 1:200 |
| Phospho-RIPK1 (Ser166) | Affinity | AF2398 | mIHC: 1:500 |
| 6*His | Proteintech | 66005-1-Ig | WB: 1:5000 |
| Flag | Servicebio | GB15939-100 | WB: 1:5000 |
| ACTA2 | Affinity | BF9212 | IF: 1:200 |

IF: immunofluorescence; WB: western blot; IP: immunoprecipitation; IHC: Immunohistochemistry; mIHC: Multiplex immunohistochemical staining
